# Novel prognostic marker LINC00205 promotes tumorigenesis and metastasis by competitively suppressing miRNA-26a in gastric cancer

**DOI:** 10.1038/s41420-021-00802-8

**Published:** 2022-01-10

**Authors:** Longtao Huangfu, Biao Fan, Gangjian Wang, Xuejun Gan, Shanshan Tian, Qifei He, Qian Yao, Jinyao Shi, Xiaomei Li, Hong Du, Xiangyu Gao, Xiaofang Xing, Jiafu Ji

**Affiliations:** 1 Key Laboratory of Carcinogenesis and Translational Research (Ministry of Education), Division of Gastrointestinal Cancer Translational Research Laboratory, Peking University Cancer Hospital & Institute, Fu-Cheng Road, Beijing, 100142 China; 2 Key Laboratory of Carcinogenesis and Translational Research (Ministry of Education), Gastrointestinal Cancer Center, Peking University Cancer Hospital & Institute, Fu-Cheng Road, Beijing, 100142 China; 3 National Institute on Drug Dependence, Peking University, North Huayuan Road, Beijing, 100191 China; 4 Department of Orthopedics, Shenzhen Institute of Translational Medicine, The First Affiliated Hospital of Shenzhen University, Shenzhen Second People’s Hospital, Shenzhen, 518025 China; 5 Department of Pathology, Peking University Cancer Hospital & Institute, Fu-Cheng Road, Beijing, 100142 China

**Keywords:** Gastric cancer, Epithelial-mesenchymal transition, Long non-coding RNAs

## Abstract

Rapid proliferation and metastasis of gastric cancer (GC) resulted in a poor prognosis in the clinic. Previous studies elucidated that long non-coding RNA (LncRNA) LINC00205 was upregulated in various tumors and participated in tumor progression. The aim of our study was to investigate the regulating role of LINC00205 in tumorigenesis and metastasis of GC. Both public datasets and our data showed that the LINC00205 was highly expressed in GC tissues and several cell lines. Notably, GC patients with high level of LINC00205 had a poor prognosis in our cohort. Mechanistically, knockdown of LINC00205 by shRNAs suppressed GC cells proliferation, migration, invasion remarkably, and induced cell cycle arrest. Based on bioinformatics prediction, we found that LINC00205 might act as a competitive endogenous RNA (ceRNA) through targeting miR-26a. The level of miR-26a had negatively correlated with LINC00205 expression and was decreased among GC cell lines, tissues, and serum samples. Our results for the first time confirmed that miR-26a was a direct target of LINC00205 and might have the potential to become a plasma marker for clinical tumor diagnosis. Indeed, LINC00205 knockdown resulted in the dramatic promotion of miR-26a expression as well as inhibition of miR-26a potential downstream targets, such as HMGA2, EZH2, and USP15. These targets were essential for cell survival and epithelial-mesenchymal transition. Importantly, LINC00205 was able to remodel the miR-26a-mediated downstream silence, which identified a new mechanism of malignant transformation of GC cells. In conclusion, this study revealed the regulating role of the LINC00205/miR-26a axis in GC progression and provided a new potential therapeutic strategy for GC treatment.

## Introduction

As the fifth most common cancer worldwide, gastric cancer (GC) is still incurable and induces about 760,000 mortalities each year [1]. What is worse, unresectable or metastatic GC is associated with poor prognosis, and systemic chemotherapeutic approaches provide minimal benefit [2–4]. Thus, novel biomarkers for improving GC, early diagnosis, prognostic evaluation, and tumor grading are urgently needed.

Due to the continuous development of high throughput sequencing technology, scientists realized that most of the human genome is transcribed into non protein-coding RNA, which indicates that a large group of RNA regulators is dedicated to modulating a relatively small number of effectors [5, 6]. Among the newly discovered RNA elements, long non-coding RNAs (lncRNAs) have been identified to function as key regulators of diverse cellular processes, such as development, differentiation, and cell fate as well as disease pathogenesis [7, 8]. LncRNAs can serve as signal mediators, molecular decoys, and scaffold or enhancers of transcription, and what is intriguing is that a large group of lncRNAs function as competing for endogenous RNA (ceRNA) that regulate gene expression through sponging miRNAs [9].

Accumulating evidence reveals that lncRNAs are important regulators of oncogenes and tumor suppressor genes, and are involved in the occurrence and development of GC [10, 11]. Until now, the functions and underlying mechanisms of several lncRNAs in GC have been reported [12–14]. However, more specific roles of lncRNAs in GC tumorigenesis and metastasis need to be explored. In this study, we determined the level of LINC00205 in tissue samples of different GC subtypes and revealed the mechanism of how LINC00205 modulates GC cells proliferation, migration, and invasion.

## Results

### LINC00205 was highly expressed in GC tissue and indicated a poor prognosis

**Fig. 1:**
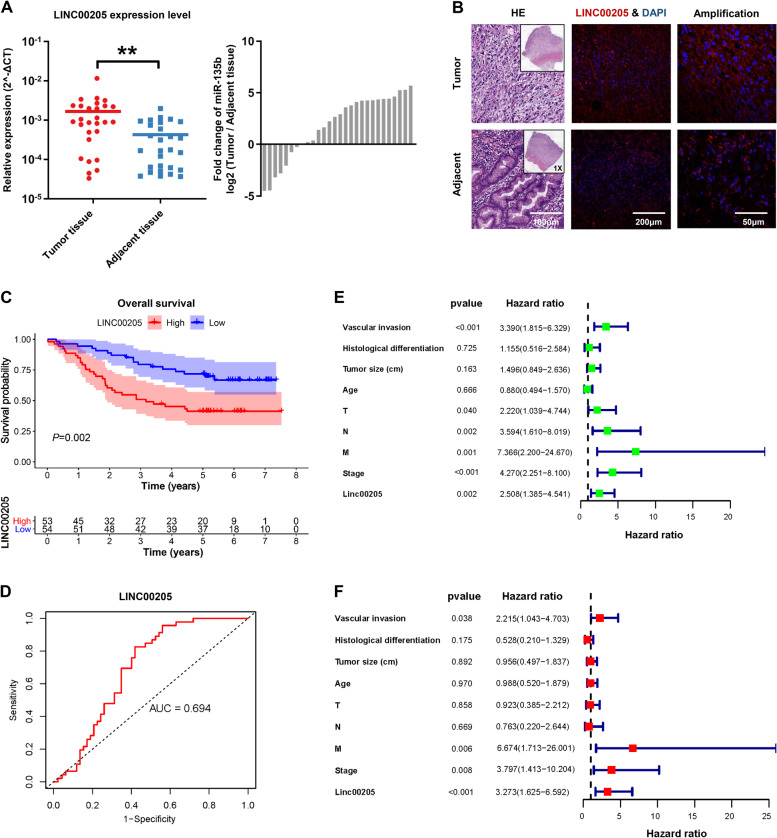
LINC00205 overexpression was strongly associated with gastric cancer (GC) pathogenesis. **A** The expression of LINC00205 in GC tissues and matched adjacent normal tissues from 28 patients in the Peking University Cancer Hospital (PUCH) cohort. **P < 0.01 calculated by paired Student’s *t*-test. **B** RNA FISH of GC tissues and adjacent tissues showing LINC00205 expression. Hematoxylin and eosin (H&E)-stained section is shown (left). Tissues imaged under fluorescence illustrating LINC00205 expression in a subpopulation of cells; high magnification is shown (right). LINC00205 probe (red) and DAPI (blue). Scale bar, 100 μm. **C** Kaplan–Meier plots for the overall survival rate of patients with GC in group of LINC00205 high or low expression levels in the PUCH cohort. *P* = 0.002 calculated by the log-rank test. **D** The time-dependent ROC curves for 5 years overall survival were used to assess the prognostic accuracy of LINC00205 expression level. Data were bootstrap-corrected AUC. **E** The univariate analysis and **F** The multivariate analysis of prognostic parameters in patients with GC by Cox regression analysis.

The clinical information and demographic characteristics of the cohort with 107 GC patients included in this study were shown in Table S1. The expression level of LINC00205 in GC samples was analyzed using quantitative real-time RT-PCR (qRT-PCR). A total of 28 paired tumors and adjacent normal tissues were examined, the results showed that LINC00205 was highly expressed in GC tissues compared with adjacent tissues (Fig. 1A), which was also confirmed among public databases (Fig. S1A, B). To further detect the expression pattern of LINC00205 in GC tissue and adjacent tissues, we performed RNA fluorescence in situ hybridization (RNA FISH) using specific probes for LINC00205 transcript (red). And, we found that the expression of LINC00205 was significantly higher in tumor tissues than in adjacent tissues (Fig. 1B). To evaluate the prognostic values of LINC00205 in the cohort of GC patients, we detected the expression level of LINC00205 in all samples and divided them into high-level group and low-level group according to the median of LINC00204 expression level. Kaplan–Meier analyses proved that patients in the low-level group had a significantly longer OS than high-level group (Fig. 1C), and time-dependent ROC curves indicated that the expression signature of LINC00205 had high predictive accuracy for 5-year OS as well (AUC = 0.694, *P* < 0.01) (Fig. 1D). In the univariate analysis, patients with a high level of LINC00205 had poorer OS than those in the low LINC00205 group (hazard ratio = 2.508, 95% confidence interval = 1.385–4.541) (Fig. 1E). In the multivariate analysis, a Cox proportional hazard model was adjusted for vascular invasion, histological differentiation, tumor size, age, TNM stage, and clinical stage. High level of LINC00205 subgroup had poorer OS than the low level subgroup (hazard ratio = 3.273, 95% confidence interval = 1.625–6.592) (Fig. 1F). These results suggested that abnormal expression of LINC00205 might be closely related to the progression of GC.

### Identification of LINC00205 at the subcellular and prediction of its function in GC progression

**Fig. 2:**
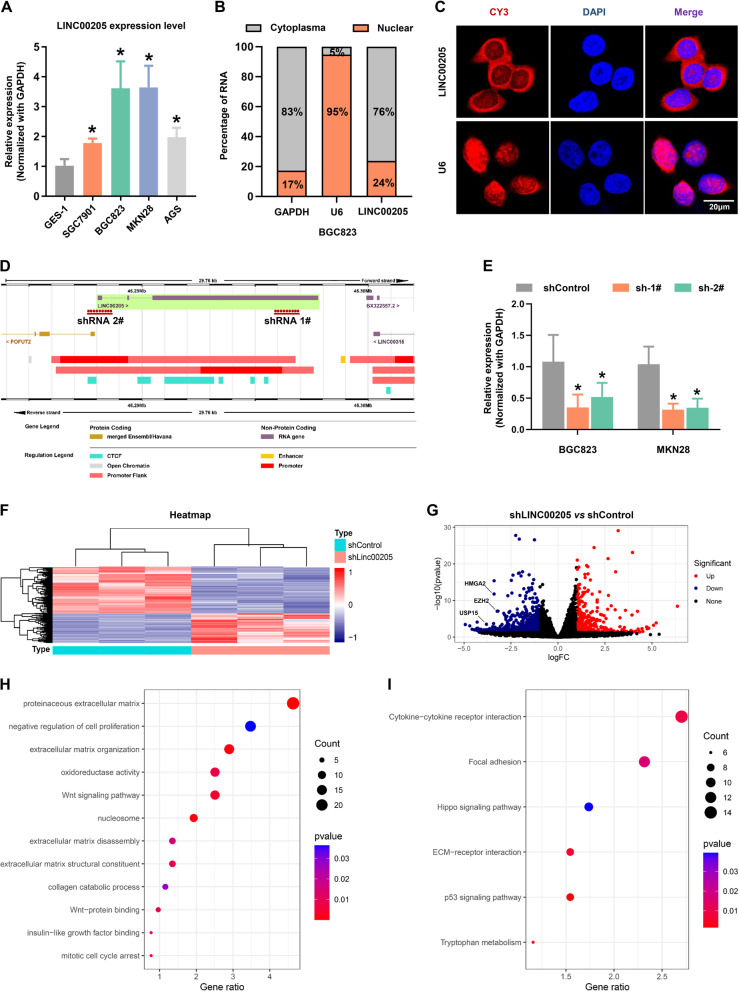
Identification of LINC00205 at the subcellular and prediction of its function in GC progression. **A** Relative LINC00205 expression levels in cell lines derived from normal gastric mucosa and primary GC by qRT-PCR analysis. *P < 0.05 *vs* GES-1. **B** Subcellular localization of LINC00205 determined by qRT-PCR in BGC823 cells. **C** Representative confocal microscopy images of RNA-FISH against LINC00205 sequence in BGC823 cells. LINC00205 probe (red) and DAPI (blue). Scale bar, 20 μm. **D** Schematic annotation of LINC00205 obtained from the Ensembl Genome Browser by BLAST search (Ensembl ID: ENSG00000223768). **E** BGC823 and MKN28 cells were transfected with LINC00205 shRNAs to establish the stable knockdown cell lines. The transfection efficiency was further confirmed by qRT-PCR. All groups were normalized to shControl. *n* = 6 independent experiments, *P < 0.05 *vs* shControl. The RNAseq analysis of BGC823-shLINC00205 group and control group. **F** Heatmap and **G** volvano plot were shown. The significant different expression genes (top 200) were further performed **H** Gene Ontology (GO) and **I** KEGG pathway enrichment analysis. Terms with their *P-value*, rich factor, and gene count.

To determine the cellular characterization of LINC00205, we detected the expression level of LINC00205 in an immortalized cell lines and several GC cell lines and found that LINC00205 expression was significantly higher in BGC823 and MKN28 cells than others (Fig. 2A). It has been known that lncRNA function can be largely affected by its subcellular localization. Accordingly, we separated the nucleus from the cytoplasm, as indicated by the detection of GAPDH mRNA exclusively in the cytoplasmic fraction, and the detection of nucleus-retained U6 predominantly in the nuclear fraction. It revealed that most of the LINC00205 were detected in the cytoplasmic fraction (Fig. 2B). RNA-FISH imaging analysis further confirmed that LINC00205 was predominantly present in the cytoplasm (Figs. 2C and S2A). Next, we annotated the genomic coordinates of LINC00205 on the Ensembl Genome Browser and found that it resides on chromosome 21 in human composing of 3 exons with a full length of 7,540 bp and it has no protein-coding potential (Fig. 2D). To further predict the molecular functions of LINC00205 in GC progression, we synthesized two shRNA sequences to knockdown LINC00205 in GC cells and further established stable cell lines. The efficiency of LINC00205 knockdown in BGC823 and MKN28 cells was detected by qRT-PCR (Fig. 2E). Then, we extracted RNA from these cells and performed transcriptome sequencing. We identified a set of differentially expressed genes after LINC00205 knockdown and presented these genes via heatmap and volcano plot, respectively (Fig. 2F, G). The result of GO and KEGG enrichment analysis showed high confidence of genes enriched in the function of cell proliferation, cell cycle, and cell adhesion, consistent with the canonical molecules of these pathways significantly modulated (Fig. 2H, I). We next performed gene set enrichment analysis (GSEA) in our database and also found similar results (Fig. S2B, C). The above results suggested that LINC00205 could affect the key regulated pathway that controls the progression and metastasis of GC.

### Knockdown of LINC00205 inhibited GC cells proliferation and suppressed tumor growth

**Fig. 3:**
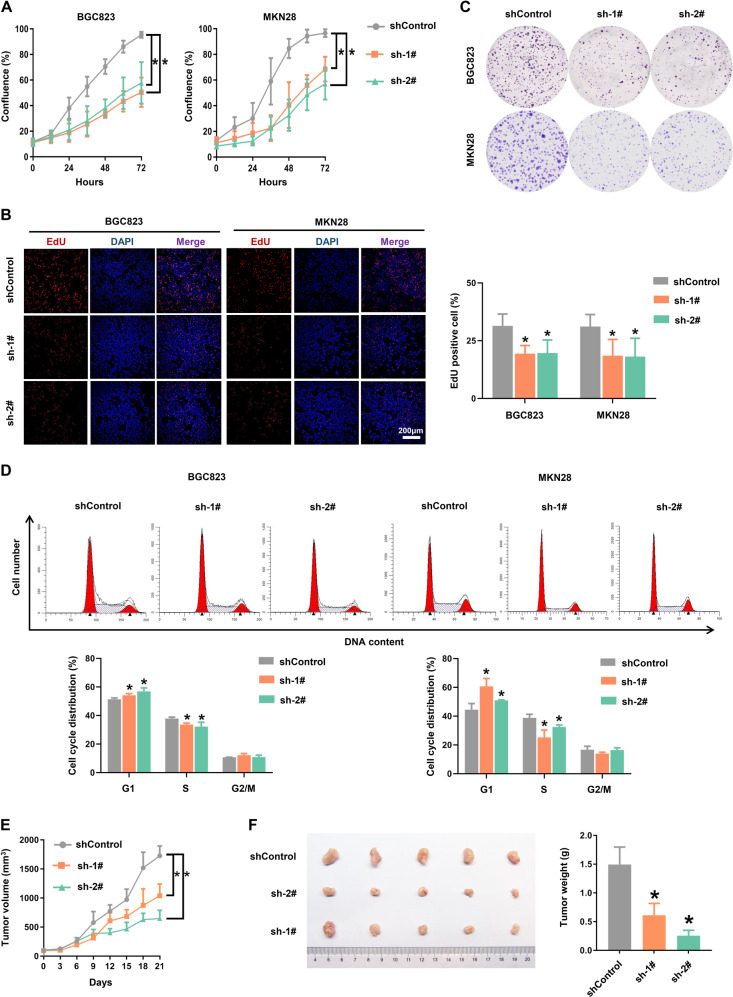
Knockdown of LINC00205 inhibited cell proliferation and tumor growth. **A** Cell proliferation was measured by IncuCyte live cell analysis system. **B** The activity of DNA replication was detected by EdU staining. The representative images were shown (left) and the quantitative measurements of the percentage of EdU positive cells were presented in column shows (right). *n* = 6 independent experiments, *P < 0.05 *vs* shControl. Scale bar, 200 μm. **C** Colony formation assay. **D** Cell cycle analysis of the indicated groups. Figure shown was a representative experiments (up). The percentage of cells in each phase was calculated (down). The BGC823 cells with stable knockdown of LINC00205 were xenograft subcutaneously into the immune-deficient nude mice (*n* = 5 for each group). The tumor volumes were calculated and recorded when the xenograft models established. **E** The tumor growth curves. *P < 0.05 *vs* shControl. At the end of experiment, the mice were sacrificed and the tumors were collected. **F** The representative images of xenograft tumors (left). Tumor weight was statistically plotted (right).

To evaluate the functions of LINC00205 on cell biological behaviors, biochemical assays were performed with LINC00205 knockdown cell lines. IncuCyte system for live-cell imaging and analysis was used to determine tumor cell proliferation activity, and knockdown of LINC00205 was consistently reduced the number of proliferating cells in both BGC823 and MKN28 cell lines, compared with the control cells after 3 days (Fig. 3A). The activity of DNA replication in LINC00205 knockdown cells was markedly decreased (Fig. 3B), which further confirmed that LINC00205 was involved in the regulation of aberrant proliferation signaling in GC cells. We also found that the colony formation abilities of cells with lower expression of LINC00205 were notably suppressed (Figs. 3C and S3A). Interestingly, knockdown of LINC00205 resulted in decreasing the percentage of cells in the S phase, whereas the cells in G1 phase were increased, compared with control cells (Fig. 3D). Besides, the expression level of the cell cycle regulation marker CyclinD1 was decreased in LINC00205 knockdown cells (Fig. S3B–D). These results suggested that a relatively larger number of cells with a low level of LINC00205 failed to go through the cell cycle smoothly to proliferate. To further establish the functional importance of LINC00205 on the progression of GC, nude mice were subcutaneously injected with BGC823 cells (BGC823-shControl, BGC823-sh1, BGC823-sh2), respectively. After 1 week, xenograft tumor models could be observed growing by eyes nearly at the same time. Similar to the results of in vitro experiments, tumors exhibited a slower growth rate and smaller size in LINC00205 knockdown cells injected group (Fig. 3E, F). These results demonstrated that LINC00205 played an important role in the occurrence and progression of GC, and inhibiting the expression of LINC00205 could suppress the tumorigenic ability of GC cells.

### Knockdown of LINC00205 reversed EMT process remodeling and inhibited GC metastasis

**Fig. 4:**
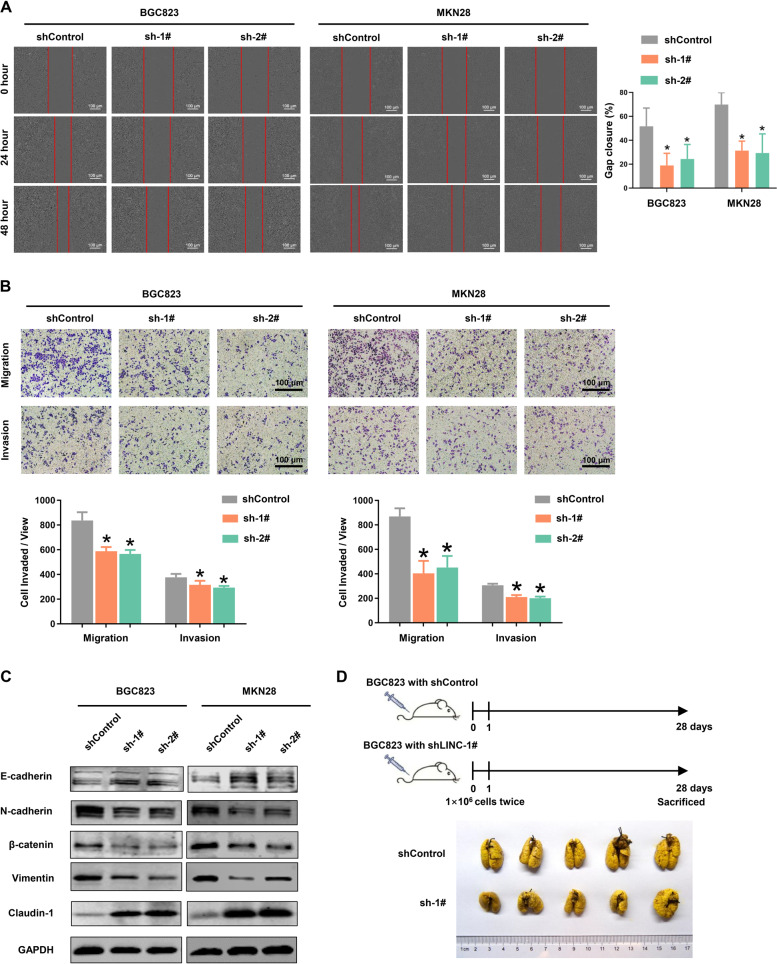
Knockdown of LINC00205 attenuated GC cell migration and invasion both in vitro and in vivo. **A** Wound-healing assay was used to determine the mobility of GC cells. **B** GC cells were further assayed for their invasive capability with or without Matrigel on transwell chambers. *n* = 6 independent experiments, *P < 0.05 *vs* shControl. Scale bar, 100 μm. **C** Western blot analysis of EMT-related proteins E-Cadherin, N-Cadherin, β-catenin, Vimentin, and Claudin-1. **D** The effects of LINC00205 on metastatic colonization through blood circulation. Knockdown of LINC00205 attenuated BGC823 cells’ colonization to the lungs.

As the high expression level of LINC00205 was associated with metastasis in patients with primary GC, we assumed that LINC00205 might endow GC cells with invasive behavior. As expected, both wound healing and matrigel invasion assays confirmed that knockdown of LINC00205 significantly inhibited GC cells migration and invasion, compared to control cells (Fig. 4A, B). Importantly, knockdown of LINC00205 partially reversed the remodeling of the epithelial-mesenchymal transition (EMT) process, as promoting the expression of an epithelial cell marker (E-cadherin) and reducing the expression of mesenchymal related markers (N-cadherin and Vimentin) (Figs. 4C and S4). Next, we evaluated the effect of LINC00205 knockdown on tumor metastatic colonization in nude mice. As usual, BGC823 cells stably transfected with empty vector and LINC00205 shRNA were injected into nude mice via the tail vein. After 6 weeks, the metastatic potential was further assessed by counting colonized tumor nodules in the lung and only slight metastasis was found in mice injected with LINC00205 knockdown cells (Fig. 4D). These data indicated that LINC00205 aggressively promoted cell migration and invasion in GC.

In contrast, overexpression of LINC00205 in SGC7901 cells promoted cell proliferation, migration, and invasion (Fig. S5). In summary, Our results suggest that LINC00205 is a potential oncogene that regulates the malignant transformation of GC cells.

### LINC00205 bound to tumor suppressor miR-26a and silenced its downstream functions

**Fig. 5:**
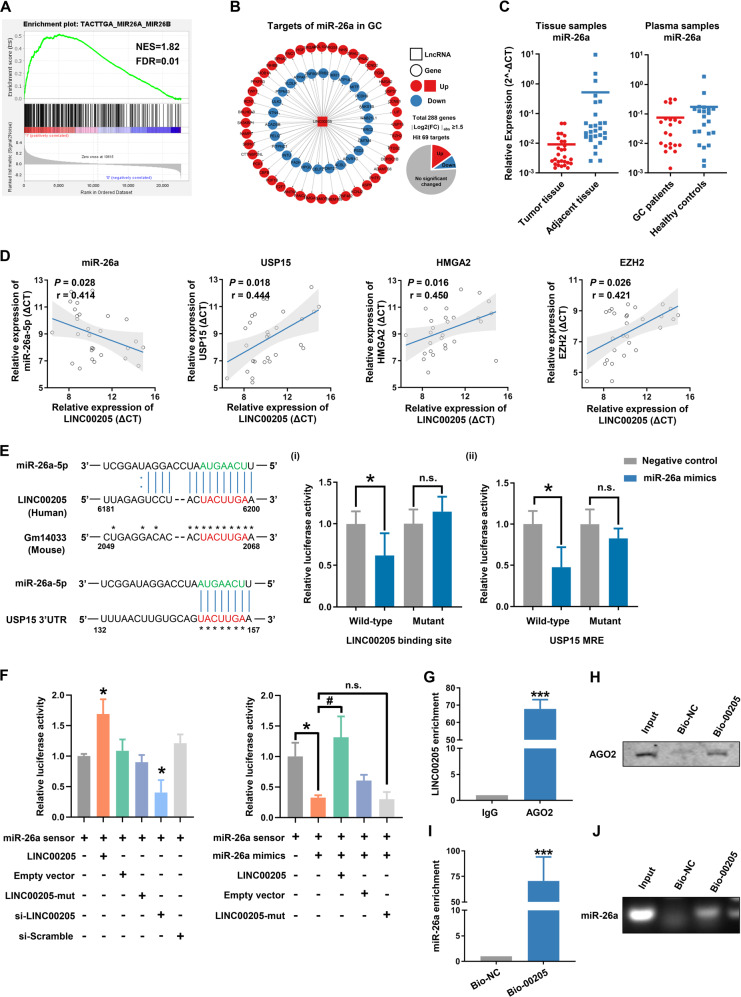
LINC00205 regulated the expression and activity of miR-26a. **A** Gene Set Enrichment Analysis (GSEA) using the miRDB subset explored the target sets of microRNAs enriched in the group of high expression of LINC00205. **B** The interaction network of miR-26a targets. USP15, EZH2, HMGA2 were the key nodes of the miR-26a regulatory gene expression network. **C** Left panel: qRT-PCR analysis for miR-26a expression in 28 paired primary GC tissues and adjacent tissues. U6 was used as an internal control. Right panel: The level of circulating miR-26a in the plasma samples. The plasma samples of GC patients were collected before operation (*n* = 21) and the healthy controls were taken from a physical examination (*n* = 22). **D** The correlation of LINC00205 among miR-26a, USP15, EZH2, and HMGA2 expression levels in GC tissues (*n* = 28). **E** Left panel: LINC00205 contained a sequence domain complementary to the seed motif of miR-26a, Gm14033(mouse) has high homology with LINC00205(human). Right panel: (I) Luciferase reporter activities of chimeric vectors carrying luciferase gene and a fragment of LINC00205 containing wild-type binding site or mutant binding site for miR-26a were detected in HEK293 cell line. (ii) USP15 is a potential target of miR-26a in humans. Luciferase reporter activities of chimeric vectors carrying either wild-type or mutant 3'UTR of USP15 were also detected in HEK293 cell line. **F** LINC00205 binds to miR-26a and regulates its activity in BGC823 cells. *n* = 6 independent experiments, *P < 0.05 *vs* miR-26a sensor. **G** The RIP assay revealed that LINC00205 was enriched by AGO2 in BGC823 cells. ***P < 0.001 *vs* IgG. **H** RNA affinity isolation in GC cells using biotin-labeled LINC00205 probes. Western blot was used to detect enrichments of AGO2. *n* = 3. **I** miR-26a was pulled down by LINC00205 probe, and the expression of miR-26a was analyzed by qRT-PCR. **J** The product of qRT-PCR was identified by agarose gel electrophoresis. Bio-NC, negative control of LINC00205.

Evidence is mounting that lncRNAs act as ceRNA (competitive endogenous RNA) that competitively bind to and restrain the activity of miRNAs. As shown in Fig. 2B, C, LINC00205 was mainly present in the cytoplasm, we inferred that it might bind to the specific miRNA to regulate cells' malignant transformation. In order to determine which miRNAs can directly bind to LINC00205, we performed GSEA to identify the targets of each miRNA that were enriched in the group of high expression levels of LINC00205 or the GC cells with LINC00205 knockdown (Figs. 5A and S6A). The candidate miRNAs were filtered based on RNAhybrid, a binding sites prediction algorithm, and the targets expression profiles of miRNAs in GC. miR-26a (miRBase Accession ID: MIMAT0000082), which was an important tumor suppressor related to EMT and stemness processes, was selected for further analysis (Figs. 5B and S6B). The qRT-PCR analysis showed that the level of miR-26a was markedly decreased in both tumor tissue samples and plasma samples of GC patients (Fig. 5C). And, knockdown of LINC00205 induced a significant increase of miR-26a expression in GC cells (Fig. S6C). We also detected the expression levels of three targets of miR-26a and found that there was a negative correlation between the expression levels of LINC00205 and miR-26a, and a positive correlation with each target of miR-26a (Fig. 5D and Fig. S6D–F). These results suggested that miR-26a was a potential binding target of LINC00205.

To further delineate the downstream signaling mechanisms responsible for the tumorigenesis effects of LINC00205, computational-analyzes identified that LINC00205 and the gene encoding USP15, which is broadly conserved among multiple species, contained the seed sequence for miR-26a (Fig. 5E, left). USP15 has previously been reported to be a key factor in tumor pathogenesis and a potential therapeutic target of cancers. Intriguingly, USP15 was found amplified in GC (Fig. S7A), and amplification conferred poor prognosis in patients with GC (Fig. S7B). The combination between miR-26a and either LINC00205 or USP15 was confirmed by the luciferase experiment, respectively (Fig. 5E, right). Subsequent experiments validated the direct regulatory effect of LINC00205 on miR-26a. A miR-26a sensor luciferase vector containing a fragment of the LINC00205 sequence that included the miR-26a binding site incorporated into the 3'-UTR of the luciferase gene was constructed. Overexpression of LINC00205 increased luciferase activity and silencing of LINC00205 had the opposite effect (Fig. 5F, left), indicating that LINC00205 could bind to miR-26a and reduce the availability of functional miR-26a. Besides, miR-26a significantly inhibited the luciferase activity of sensor vector, inhibition which was alleviated by co-expression of LINC00205. The mutated LINC00205 (LINC00205-mut), which lacked the miR-26a binding site, failed to alleviate inhibition of luciferase activity by miR-26a (Fig. 5F, right).

Next, we performed RNA immunoprecipitation (RIP) using AGO2 antibody, a component of RNA-induced silencing complex (RISC) to mediate miRNA-induced gene silencing. qRT-PCR analysis showed that LINC00205 was enriched in the RISC (Fig. 5G). In order to test whether LINC00205 could pull down miR-26a, we constructed a biotin-labeled LINC00205-specific probe and performed an RNA affinity-isolation assay. In line with the result of the RIP experiment, LINC00205 directly interacted with AGO2 (Figs. 5H and S8), whereas qRT-PCR analysis and agarose gel electrophoresis further confirmed the binding of LINC00205 and miR-26a (Fig. 5I, J). Taken together these results suggest that LINC00205 interacted directly with miR-26a and AGO2 to form RISC, and regulate the expression and activity of miR-26a.

## Discussion

Although improvements of clinical therapies have been achieved to improve the clinical condition, GC patients with advanced stages and distant metastasis still have very poor prognoses, making it urgent to find new targets against GC cell proliferation and metastasis [15]. Recently, a great number of lncRNAs have been identified to participate in tumor progression and gain much attention [16, 17]. LINC00205 is a newly discovered non-coding RNA showing a strong association with prognosis according to our preliminary study on lncRNA expression profile. However, the biological functions and molecular mechanisms of LINC00205 in GC have been unknown yet. In this study, we identified LINC00205 as an important target for treatment in GC progression. Functional in vitro and in vivo experiments showed that knockdown of LINC00205 suppressed GC cell proliferation, migration, invasion, and induced cell cycle arrest, whereas overexpression of LINC00205 led to the opposite. Importantly, we discovered a novel mechanism by which LINC00205 promoted GC progression and paved the way for subsequent clinical studies.

MicroRNAs (miRNAs) are endogenous non-coding RNAs with a length around 21–23 nt. miRNAs function by binding to argonaute (AGO) protein and constitute RISC. This complex could be tethered to complementary mRNAs, resulting in mRNA degradation [18, 19]. Our present work focused on miR-26a, which has been shown to be frequently downregulated in a variety of malignancies and act as a potential tumor suppressor. miR-26a has been validated to be downregulated in multiple cancer types and overexpression of miR-26a might suppress the malignant phenotypes of tumor cells by targeting several oncogenes [20–22]. However, there are few studies that reported the upstream regulatory pathway of miR-26a, and the mechanism of endogenous inhibition effect of miR-26a during tumor progression has been unclear.

It has been well demonstrated that lncRNAs participate in multiple human diseases by acting as sponges to reduce the expression levels of miRNAs and correspondingly increase the levels of target genes [23, 24]. The lncRNA HOXD-AS1 is reported to facilitate metastasis of liver cancer by competitively bound to miR-130a-3p that prevented SOX4 from miRNA-mediated degradation [25]. The lncRNA PAGBC has been found to competitively bind to the tumor-suppressive microRNAs miR-133b and miR-511 [26]. Our previous study demonstrated that LINC01503 could epigenetically silence DUSP15/CDKN1A expression to enhance GC tumorigenesis [27]. Besides, we also found lncRNA TSPEAR-AS2 drived GC progression through regulating GJA1 and CLDN4 expression [28]. These reports indicated that lncRNAs can interact with miRNAs to exert biological functions. We identified for the first time that LINC00205 was a distinct ceRNA for miR-26a in the homo sapiens. LINC00205 bound to miR-26a, resulting in the RISC complex failed to silence the targets of miR-26a. It is not likely that ceRNA regulation is the only mechanism by which lncRNAs affect levels of miRNAs. We concluded that LINC00205 regulated miR-26a only partially through ceRNA, but the other mechanisms of miR-26a inhibition by LINC00205 need to be studied further.

Generally, the expression level of lncRNAs is much lower among tissues, but the biological functions of lncRNAs are not limited by their low expression [29]. We found that LINC00205 regulated the miR-26a-induced target expression silence, likely due to competition with target genes for the binding site of miR-26a. LINC00205 promoted the proliferation of GC cells via modification of cell cycle checkpoints and conduced to metastasis through EMT process, which might be mainly caused by the repression of miR-26a.

In the present study, we demonstrated that the high expression level of LINC00205 was observed in GC tissues and cell lines, suggesting a critical role of LINC00205 in GC. Although the tiny association between LINC00205 expression and clinicopathological characteristics of GC patients, Kaplan–Meier analysis showed that high expression of LINC00205 was positively correlated with poor survival in GC patients. In addition, multivariate analysis revealed the expression level of LINC00205 as an independent prognostic factor for the OS of GC patients. Thus, our observations combined with clinical data strongly suggested that LINC00205 might be a valuable biomarker for GC. Nevertheless, because GC is a complex disease, larger-scale mechanistic studies are warranted to confirm the real contribution of LINC00205 to GC in Chinese populations or to investigate the association between LINC00205 and different tumors in different ethnicities, limiting the confounding effect of population heterogeneity.

In short, the present work identified a novel prognostic biomarker LINC00205, which promoted GC progression and metastasis by modulating miR-26a levels via a competitive inhibition mechanism. Thus, adjusting expression of LINC00205 may provide a novel approach for the diagnosis and treatment of GC.

## Materials and methods

### Clinical specimen collection

A number of 107 GC tissue samples and 28 pairs of GC tissue samples and adjacent normal tissue samples were obtained from GC patients who underwent surgical treatment without preoperative radiotherapy and/or chemotherapy at the Peking University Cancer Hospital. The specimens were snap-frozen in liquid nitrogen and stored at −80 °C until use. The histology and age information are included in Supplementary Table S1.

Blood samples from 21 GC patients and 22 health controls were collected as previously described [30]. The histology and age information are included in Supplementary Table S2.

All patients involved in this study have written informed consent, in compliance with the Declaration of Helsinki. The Ethics Committee of the Peking University Beijing Cancer Hospital has approved this clinical specimen study (Permission number 2019KT111).

### Cell lines

The GC-derived cell lines SGC7901, BGC823, MKN28, and AGS, and the normal gastric mucosa-derived cell line GES-1 were purchased from the Chinese National Infrastructure of Cell Line Resource. All of these cell lines were recently authenticated by STR profiling and tested for mycoplasma contamination. The details of the cell culture and the establishment of ectopic expression or knockdown cell lines procedures can be found in the Supplemental Methods.

### RNA fluorescence in situ hybridization

The RNA fluorescence in situ hybridization (RNA-FISH) analysis was used to detect the subcellular location of LINC00205 using FISH Detection Kit (RiboBio, Guangzhou, China) in both GC tissues and cell lines. Fluorescence-labeled probes specially for LINC00205 (red) and DAPI (blue) for nuclear detection were used. Images were captured using LSM 800 confocal microscope (Carl Zeiss, Jena, Germany).

### Subcellular fractionation

For nuclear and cytoplasmic fraction separation, RNAs from BGC823 cells were isolated by using the PARIS kit (Life Technologies, USA) according to the manufacturer’s instructions. The RNA expression level of LINC00205 in nuclear and cytoplasmic fractions was determined by qRT-PCR. U6 was a positive control for nuclear fraction and GAPDH was a positive control for a cytoplasmic fraction.

### RNA immunoprecipitation

We performed RIP experiments using Magna RIP RNA-Binding Protein Immunoprecipitation Kit (Millipore, Billerica, MA, USA) according to the manufacturer’s instructions. Antibodies for AGO2 (Proteintech) were diluted at 1:200. Total RNAs (input controls) and isotype controls (IgG) were assayed simultaneously.

### RNA affinity-isolation assay with biotinylated probe

The biotinylated probe complementary to LINC00205 was synthesized and dissolved in 500 ml of wash/binding buffer (0.5 M NaCl, 20 mM Tris- HCl, pH 7.5, 1 mM EDTA). The probes were incubated with streptavidin-coated magnetic beads (Pierce, 88816) at 25 °C for 2 h to generate probe-coated magnetic beads. GC cells lysates were incubated with probe-coated beads, and, after washing with the wash/binding buffer, the RNA complexes bound to the beads were eluted and extracted for qRT-PCR analysis. The probe sequences are included in Supplementary Table S3, and the primers used are listed in Supplementary Table S4.

### Luciferase reporter analysis

USP15 3′-UTR containing the conserved miR-26a binding sites and mutated 3′-UTRs were synthesized by Invitrogen. The fragment was subcloned into the SacI and HindIII sites downstream of the luciferase gene in the pMIR Reporter. HEK293 cells were co-transfected in 24-well plates with USP15 wild-type or mutant constructs and miR-26a mimics, negative control. The miR-26a sensor reporter was constructed according to the method previously described. Briefly, the human genomic sequence flanking pre-miR-26a was inserted into the pGL3 vector in reverse orientation downstream of the luciferase gene coding region. GC cells were co-transfected with miR-26a sensor and si-LINC00205 / LINC00205 / miR-26a mimics or their negative control using the Lipofectamine 2000 reagent. After 48 hours of transfection, the cells were harvested and lysed. Luciferase activity was assayed using the Dual-Luciferase Reporter Assay System according to the manufacturer’s instructions. Firefly luciferase values were normalized to Renilla, and relative ratios of Firefly to Renilla activity was reported.

### Statistical analysis

All the data were expressed as the mean ± standard deviation (SD). GraphPad Prism 8.0 was used for our statistical analysis. For two-group comparisons, Student’s *t*-test was used. For multiple group comparisons, one-way ANOVA was used with Bonferroni post-test for comparisons between selected two groups as well as Dunnett post-test for comparisons among all other treatment groups to the corresponding control. The survival curves were drawn by Kaplan–Meier analysis, and the log-rank test was used to compare the survival differences.

More detailed methods can be found in the Supplemental Information.

## Supplementary information

Supplementary materials

all of the co-authors’ consent letters

## Data Availability

The authors confirm that the data supporting the findings of this study are available within the article and its Supplementary materials. Raw data that support the findings of this study are available from the corresponding author, upon reasonable request.
