# Supplementary material for: Novel prognostic marker LINC00205 promotes tumorigenesis and metastasis by competitively suppressing miRNA-26a in gastric cancer
Source: Cell Death Discov. 2022 Jan 10;8:5. doi: 10.1038/s41420-021-00802-8 (PMC8748761; doi:10.1038/s41420-021-00802-8)
Supplement: Supplementary file 1 — Supplementary materials [file 41420_2021_802_MOESM1_ESM.docx]

**Supplementary Methods**

Cell culture

GC cells were seeded in Roswell Park Memorial Institute 1640 medium (RPMI 1640; GIBCO, USA), and GES-1 cells was seeded in Dulbecco’s Modified Eagle medium (DMEM; GIBCO, USA). All mediums were supplemented with 10% fetal bovine serum (FBS; GIBCO, USA). All cells were cultured at 37°C with 5% CO_2_ in a humidified incubator.

Establishment of stable LINC00205 knockdown or overexpression cell lines

The shRNAs of human LINC00205 lentivirus vector were obtained from GenePharma. Using a three-plasmid transient co-transfection method (Lenti-T HT packaging mix), replication-defective vesicular stomatitis virus-G-pseudotyped viral particles were packaged in HEK293T cells. Lentivirus-containing supernatant was harvest at 48 hrs post-transfection, purified by centrifugation and stored at −80°C. For viral transductions, 1 mL of the scrambled shControl or shLINC00205 lentiviruses were incubated with BGC823 and MKN28 cells overnight at 37°C in the cell culture incubator. Stable knockdown of LINC00205 were selected with puromycin (0.8 μg/mL) in the culture media. After the establishment of stable knockdown cell lines, we preserved these cell lines in liquid nitrogen. All experiments in this research were performed in 3-6 generations of these cell lines to ensure the knockdown efficiency.

The full length of human LINC00205 was cloned into pcDNA3.1 (+) vector to generate pcDNA3.1-LINC00205 vector. SGC7901 cells were seeded in six-well plates at 70% confluence before transfection. Transfections were performed using Lipofectamine 2000 according to the manufacturer’s instructions. After 48 hrs post-transfection, G418 was added at a concentration of 0.8 mg/mL to establish stable cell line of SGC7901 with LINC00205 overexpression.

RNA sequencing analysis

Total RNA from the BGC823 cells with LINC00205 knockdown and negative control were isolated using RNeasy mini kit (Qiagen, Germany). Paired-end libraries were synthesized by using the TruSeq RNA Sample Preparation Kit (Illumina, USA) following TruSeq RNA Sample Preparation Guide. Briefly, The poly-A containing mRNA molecules were purified using poly-T oligo-attached magnetic beads. Purified libraries were quantified by Qubit 2.0 Fluorometer (Life Technologies, USA) and validated by Agilent 2100 bioanalyzer (Agilent Technologies, USA) to confirm the insert size and calculate the mole concentration. Cluster was generated by cBot with the library diluted to 10 pM and then were sequenced on the Illumina HiSeq X-ten (Illumina, USA). The library construction and sequencing was performed at Shanghai Biotechnology Corporation. STAR (version:2.7.6a) was used to map the cleaned reads to the human GRCh38 reference genome with two mismatches 1. Then ,we ran Subread/featureCounts (version:2.0.2) with a reference annotation to generate gene expression counts and FPKM values for known gene model. All the sequencing data have been deposited at the GEO database with the accession number, GSE186732.

Functional assay *in vivo*

Female BALB/c nude mice (Five-weeks old) were purchased from Beijing Vital River Laboratory Animal Technology Co. Ltd. All animal experiments were conducted in accordance with the Institutional Animal Care and Use Committee guidelines at Peking University Beijing Cancer Hospital. We implemented the "3Rs" principle of Replace, Reduce, and Refine, advocated experimental animal welfare, and used the smallest statistically significant sample size in this study. Subcutaneous xenograft tumor model was used to estimate the effects of LINC00205 knockdown on tumorigenicity *in vivo*. The mice were randomly divided into three groups (n=5): LINC00205 sh-1#, sh-2#, and shControl. Each 2×10^6^ LINC00205 sh-1#, sh-2# or shControl BGC823 cells were resuspended in PBS with 50% Matrigel and engrafted subcutaneously into flanks of nude mice. The tumors observed in mice were measured every 3 days for three weeks. The tumor volume was calculated according to the formula: length×width^2^/2. At the end of experiment, the mice were sacrificed and the tumors were collected. For experimental tumor metastatic model, the mice were randomly divided into two groups (n=5): LINC00205 sh-1# and shControl. LINC00205 sh-1# or shControl BGC823 cells (5×10^6^ cells in a 100 μL volume per mouse) were injected into the tail vein of BALB/c nude mice. Four weeks later, the mice were sacrificed and the lungs were excised from the body. Bouin’s solution was injected from the main bronchi to fix the lung tissues.

Western blot analysis

We obtained protein samples from GC tissues and cells using RIPA lysis buffer. The samples were separated through a 12.5% SDS-PAGE and the NC membranes were blocked with 5% skim milk in at room temperature for 1 hour. Next, we incubated the membranes overnight at 4°C with primary antibodies, and GAPDH (Proteintech, #60008–1-Ig) or α/β-Tublin (CST, #2148) was used as a loading control. The primary antibodies used in this study were obtained as follow: the EMT marker antibody kit (include E-cadherin, N-cadherin, Vimentin, β-catenin, Claudin-1) (CST, #9782), the cell cycle regulation antibody kit (include CyclinD1, CDK4, CDK6) (CST, #9932), USP15 (CST, #66310), EZH2 (CST, #5246), AGO2 (Proteintech, #66720-1-Ig). The secondary antibodies, IRDye 800CW goat anti-mouse (LI-COR Biosciences , #926-32210) and donkey anti-rabbit (LI-COR Biosciences, #926-32213). The immunoreactivity was detected using Odyssey Infrared Imaging System (Gene Company Limited, China). The bands were quantified by measuring the band density.

RNA extraction and qRT-PCR

Total RNA was extracted from tissue samples and cell lines using TRIzol Reagent (Invitrogen, USA). according to the manufacturer’s instructions. First-strand cDNA was generated by RT-PCR using a reverse transcription system kit (Invitrogen, USA). qRT-PCR was performed with the ABI PRISM 7500 Sequence Detection System according to the SYBR Green method. All reactions were performed at least three times. Cycle threshold (CT) values were determined using fixed threshold settings after completion of the reaction. U6 small nuclear RNA was used as an internal control for miR-26a (For blood samples, let-7e was used as an internal control for miR-26a), and the mRNA levels of USP15, HMGA2, EZH2 were normalized to those of GAPDH. Their relative expression levels normalized to the control were further calculated using the 2^ΔΔ-ct^ method. The primers used are listed in Supplementary Table S4.

Colony formation assay

For the colony formation assay, BGC823 and MKN28cells were cultured into 6-well plates at 600 cells/well and incubated at 37°C with 5% CO_2_ for two weeks. Then, GC cells were washed three times with PBS and fixed with methanol for 15 minutes at room temperature. The cells were stained with 0.1% crystal violet, and the colonies containing >100 cells were count.

EdU staining and cell proliferation assay

BGC823 and MKN28 cells were treated as indicated, washed three times with PBS, and then incubated in serum-free RPMI 1640 containing 10 μmol/L EdU (RiboBio, China) for 2 hours. Cells were fixed, then underwent Apollo staining and nuclear staining, according to the manufacturer’s instructions. Finally, the cells were imaged by confocal microscope and the percentage of proliferating cells was further calculated.

To further determine the level of cell proliferation, the cells were seeded in 96-well plates at a density of 3×10^3^/well, then monitored with IncuCyte live cell analysis imaging system (Essen Biosciences, USA).

Cell migration and invasion assay

We tested the capacity of GC cells migration and invasion using a transwell chambers (8 μm pore size membranes). The lower chamber was added with 10% FBS and incubated at 37°C with 5% CO_2_. Then the upper surface with matrigel (BD Biosciences, USA) was used for cell invasion. And cell migration assay was conducted without matrigel. After the transfection of GC cells for 36 hours, these cells were seeded in the upper chamber with serum-free medium. 48 hours later, the migrated or invasive cells were fixed with methanol and stained with 0.1% crystal violet. We obtained images of migrated cells by using a microscope, and the cell numbers of migration and invasion were counted by using Image Pro Plus.

Bioinformatics analysis

Gastric cancer gene expression data were downloaded from The Cancer Genome Atlas (TCGA) and the Gene Expression Omnibus (GEO) dataset GSE99416. The Peking University Cancer Hospital Gastric Cancer Transcriptome Dataset (PUCH dataset) can be licensed for use by contacting the author (DOI: 10.21147/j.issn.1000-9604.2019.05.07). The data analysis was performed with R software using the DEGseq, ComplexHeatmap, clusterprofiler, tidyverse and ggplot2 packages. The target genesets of microRNAs were obtained from the website (https://www.gsea-msigdb.org/gsea/msigdb/index.jsp). The direct binding site between LINC00205 and miR-26a was predicted by RNAhybrid algorithm (<http://bibiserv.techfak.uni-bielefeld.de/rnahybrid/>). The microRNA response elements (MRE) in the mRNA 3’UTR of miR-26a targets were further confirmed by TargetScan V7.2 (<http://www.targetscan.org/vert_72/>). All data analyses were performed in the R programming and JAVA environment.

**
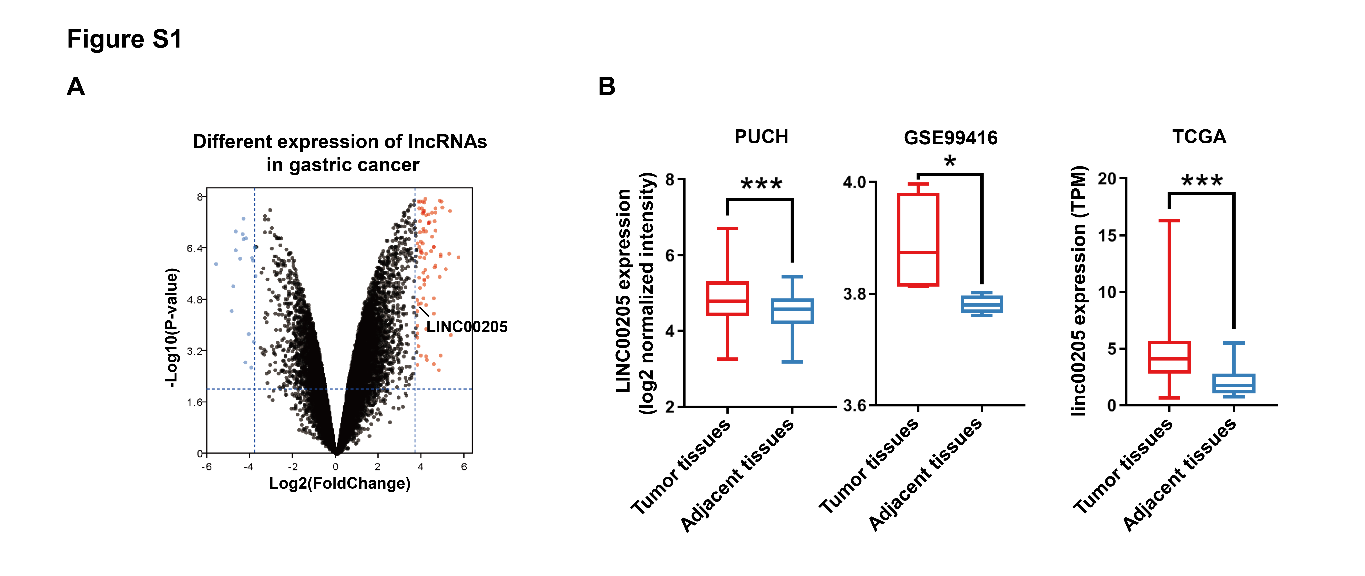
Supplementary Data**

Figure S1. Mining the expression and the potential functions of LINC00205 in public databases. (A) The expression data of lncRNAs extracted from the PUCH dataset were plotted as volcano plot. The significantly up-regulated lncRNAs in GC tissues were labeled in red and down-regulated lncRNAs were labeled in blue. The LINC00205 was indicated with the arrow. (B) The up-regulation of LINC00205 expression level was found in tumor tissues among public datasets.


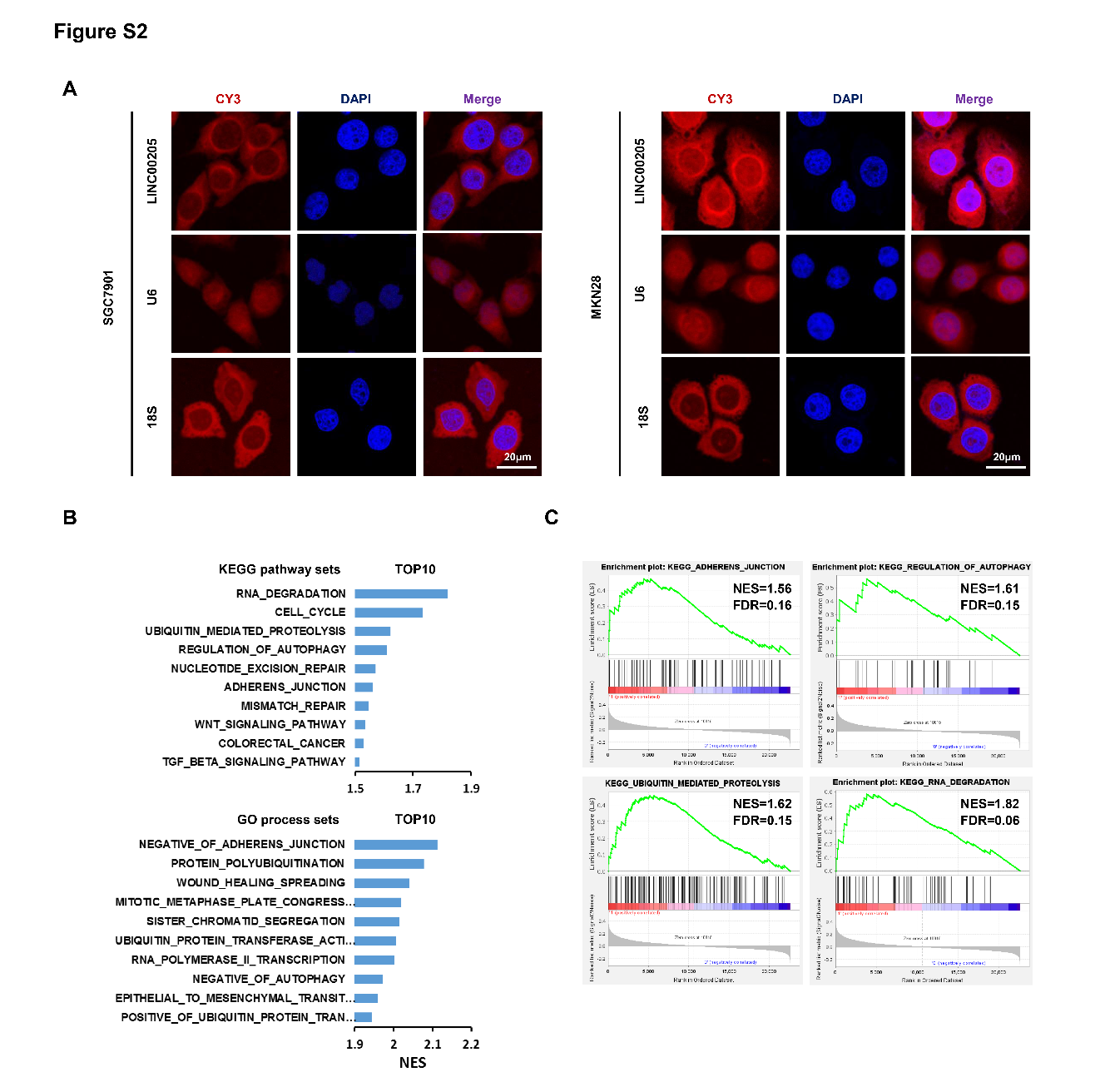


Figure S2. (A) Representative confocal microscopy images of RNA-FISH against LINC00205 sequence in SGC7901 cells (left panel) and MKN28 cells (right panel). LINC00205 probe (red) and DAPI (blue). Scale bar, 20 μm. (B) The top ten NES-ranked pathways were selected based on GSEA enrichment results. Upper: KEGG signaling pathway dataset, which are RNA degradation, cell cycle, ubiquitin-mediated proteasome degradation, cell adhesion, TGF-β pathway, etc. Lower: GO biological process datasets for negative regulation of cell adhesion, protein ubiquitination, EMT, etc., respectively. (C) GSEA analysis plots for cell adhesion, autophagy regulation, ubiquitination, and RNA degradation, respectively.


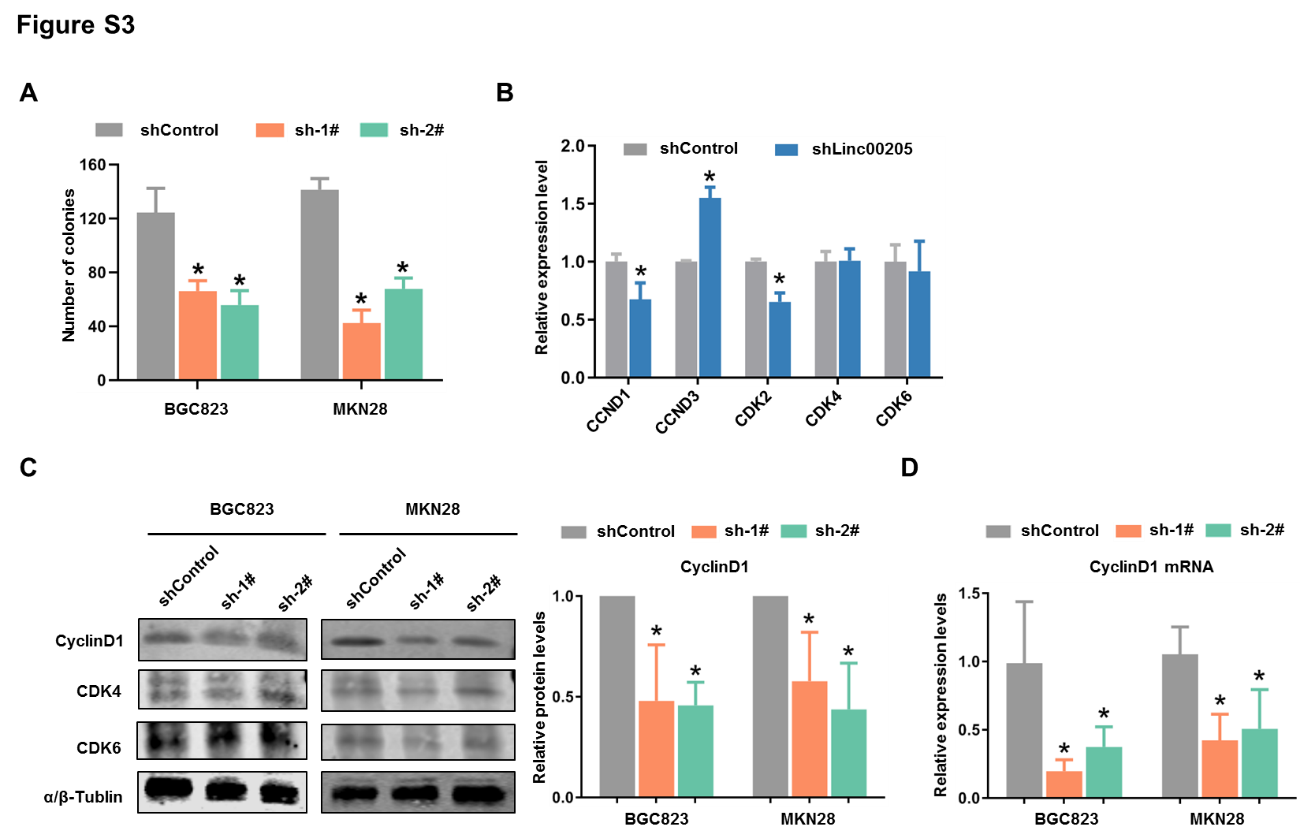


Figure S3. Knockdown of LINC00205 induced cell cycle arrest in GC cells and the statistical analysis of colony formation. (A) The statistical analyses of the colony formation assays in Figure 3C. (B) The expression levels (FPKM) of the cell cycle regulation markers were calculated from the RNAseq analysis and further normalized to shControl. The expression of CCND1 and CDK2 were significantly decreased in LINC00205 knockdown cells. (C) and (D) Western blot and qRT-PCR analyses were further performed to detect the protein and mRNA expression levels of the cell cycle regulation markers. These results suggested that knockdown of LINC00205 induced cell cycle arrest, at least in part, by inhibiting the expression of cyclin D1. α/β-Tublin or GAPDH was used as a loading control. All groups were normalized to shControl or Empty vector. n=3 independent experiments, **P* < 0.05 *vs* shControl or Empty vector.


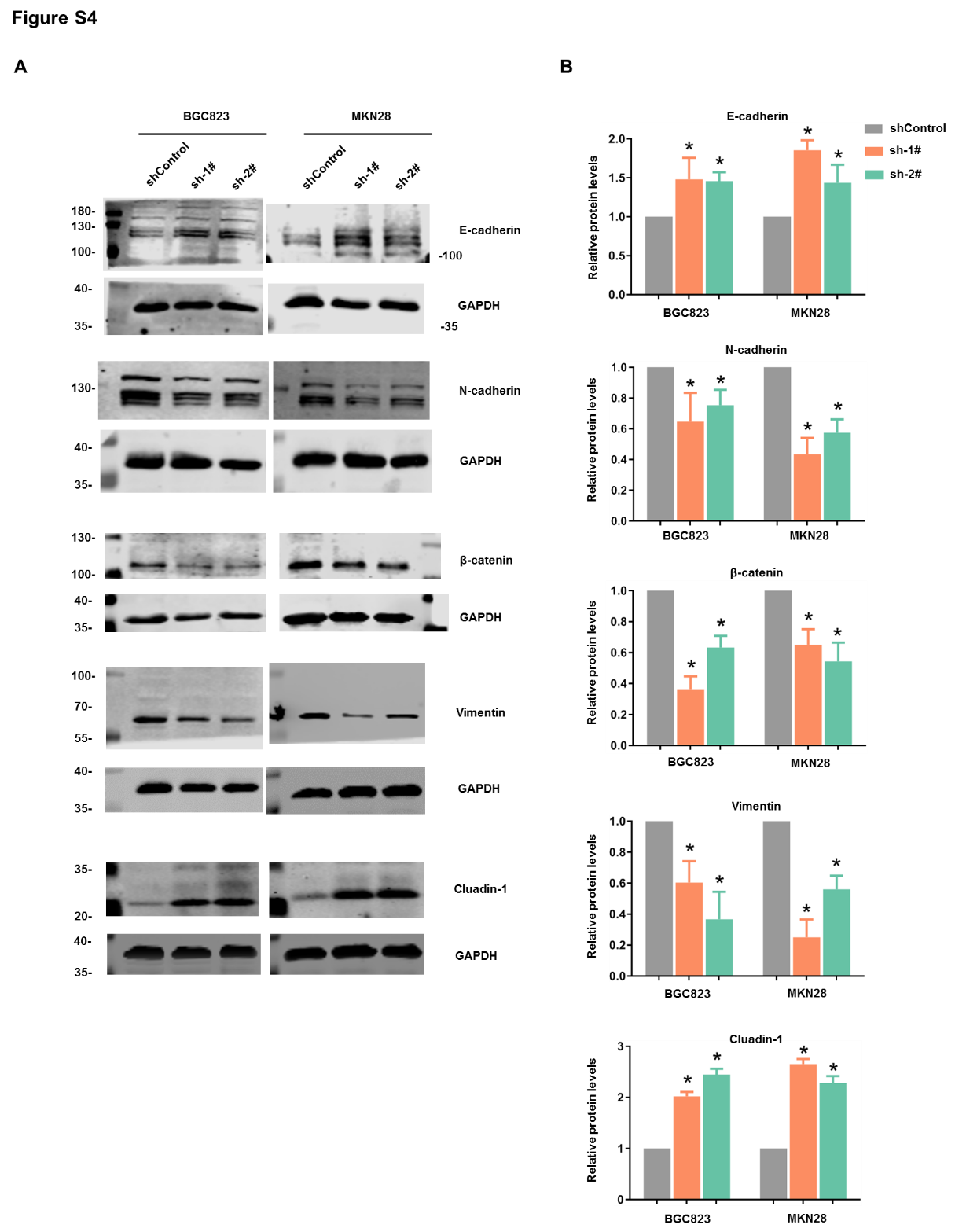


Figure S4. The original images of the bands of western blot in the main text. The protein molecular weights are marked by prestained protein ladder (#26616, Thermo, USA ). (A) Western blot analysis of EMT-related proteins E-Cadherin, N-Cadherin, β-catenin, Vimentin, and Claudin-1. GAPDH was used as a loading control. (B) The relative expression levels of EMT-related protein were calculated by Image Studio (Ver 5.2), and were statistically plotted, respectively. shControl was normalized to 1. n=3 independent experiments, **P* < 0.05 *vs* shControl.


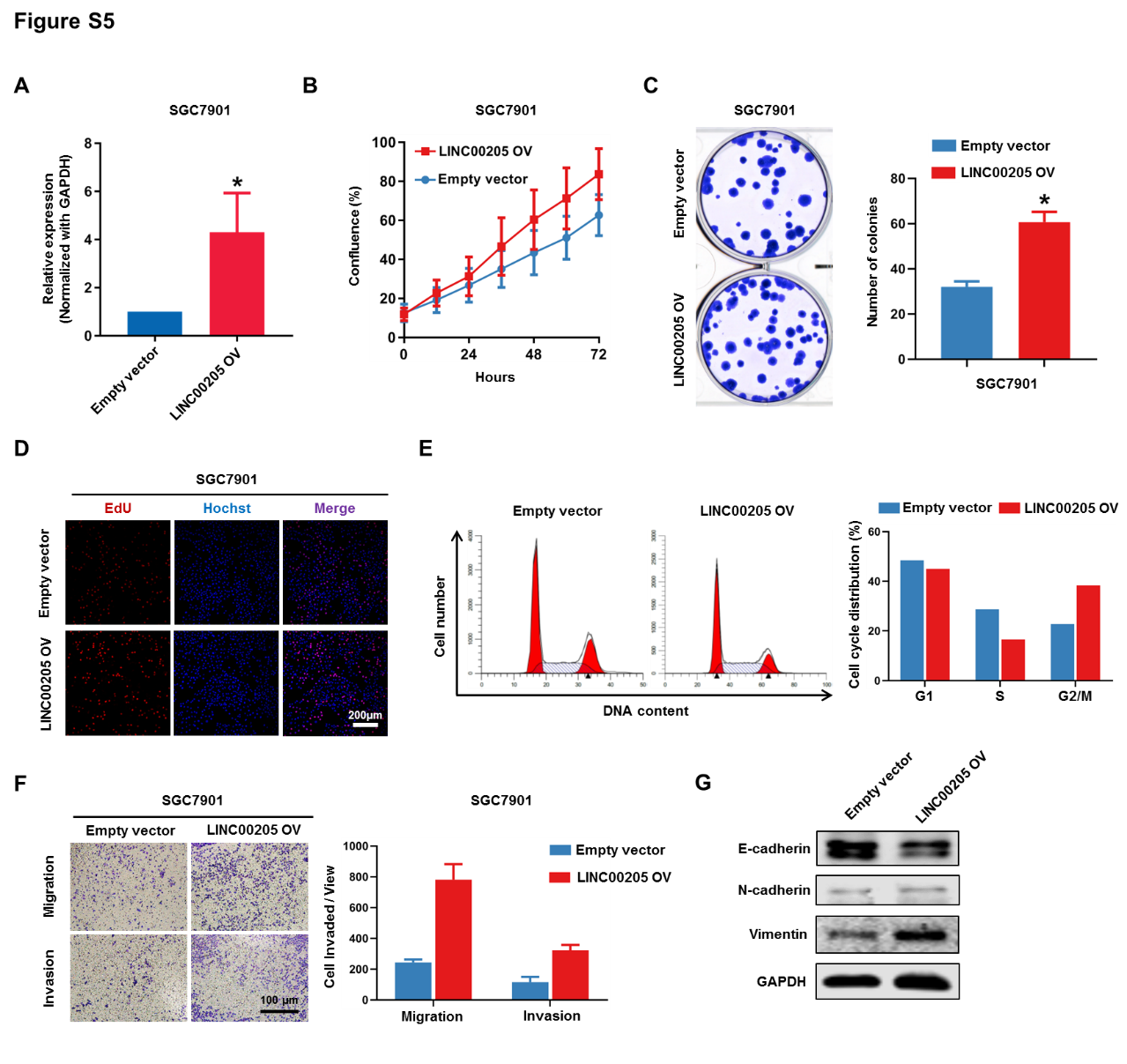
Figure S5. Overexpression of LINC00205 in SGC7901 cells promoted cell proliferation, migration, and invasion. (A) SGC7901 cells were transfected with LINC00205 overexpression plasmid to establish the stable ectopic expression cell line. The transfection efficiency was further confirmed by qRT-PCR. (B) Cell proliferation was measured by IncuCyte live cell analysis system. Overexpression of LINC00205 promoted cell proliferation. (C) Colony formation assay. Overexpression of LINC00205 enhanced the ability of colony formation in GC cells. (D) The activity of DNA replication was detected by EdU staining. Overexpression of LINC00205 increased the activity of DNA replication. (E) Cell cycle analysis of the indicated groups. Figure shown was a representative experiments (left). The percentage of cells in each phase was calculated (right). (F) GC cells were assayed for their invasive capability with or without Matrigel on transwell chambers. n= 3 independent experiments, **P* < 0.05 *vs* Empty vector. Scale bar, 100 μm. (G) Western blot analysis of EMT-related proteins E-Cadherin, N-Cadherin, and Vimentin.


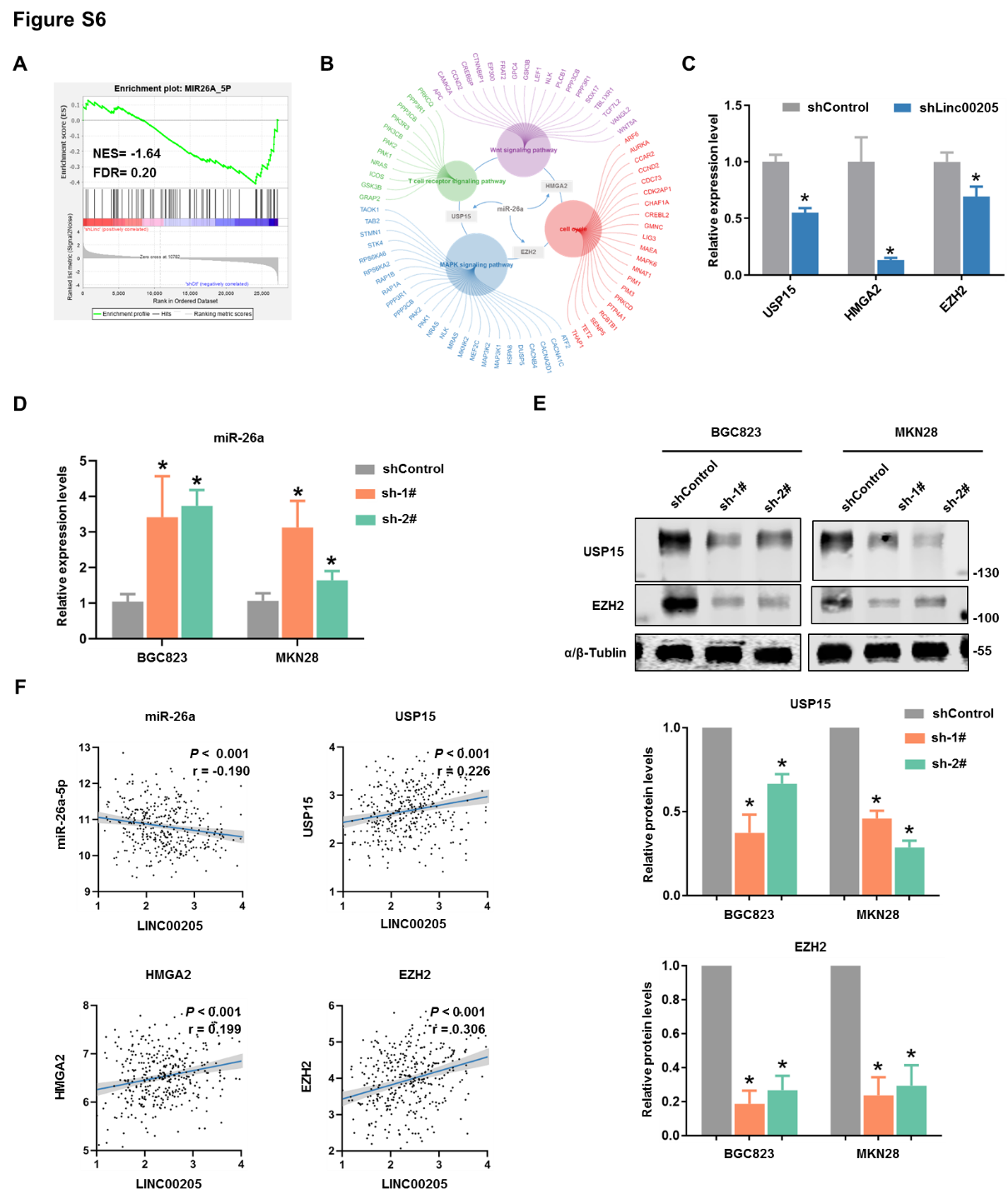


Figure S6. (A) Gene Set Enrichment Analysis (GSEA) using the miRDB subset explored the target sets of microRNAs enriched in response to LINC00205 knockdown. (B) The interaction network of miR-26a targets. USP15, EZH2, HMGA2 were the key nodes of the miR-26a regulatory gene expression network. (C) qRT-PCR analysis showed that the expression level of miR-26a was markedly decreased after GC cells transfected with LINC00205 shRNA. (D) The expression levels (FPKM) of USP15, HMGA2, EZH2 were calculated from the RNAseq analysis and further normalized to shControl. The expression levels of these targets were significantly decreased in LINC00205 knockdown cells. (E) The protein expression levels of both USP15 and EZH2 were detected by western blot analysis. α/β-Tublin was used as a loading control. All groups were normalized to shControl. n=3 independent experiments, **P* < 0.05 *vs* shControl. (F) Correlation analysis of LINC00205 and its predicted targets using TCGA data. The results showed that the expression levels between miR-26a and LINC00205 were significantly negatively correlated. In contrast, the expression levels between each target of miR-26a and LINC00205 were positively correlated.


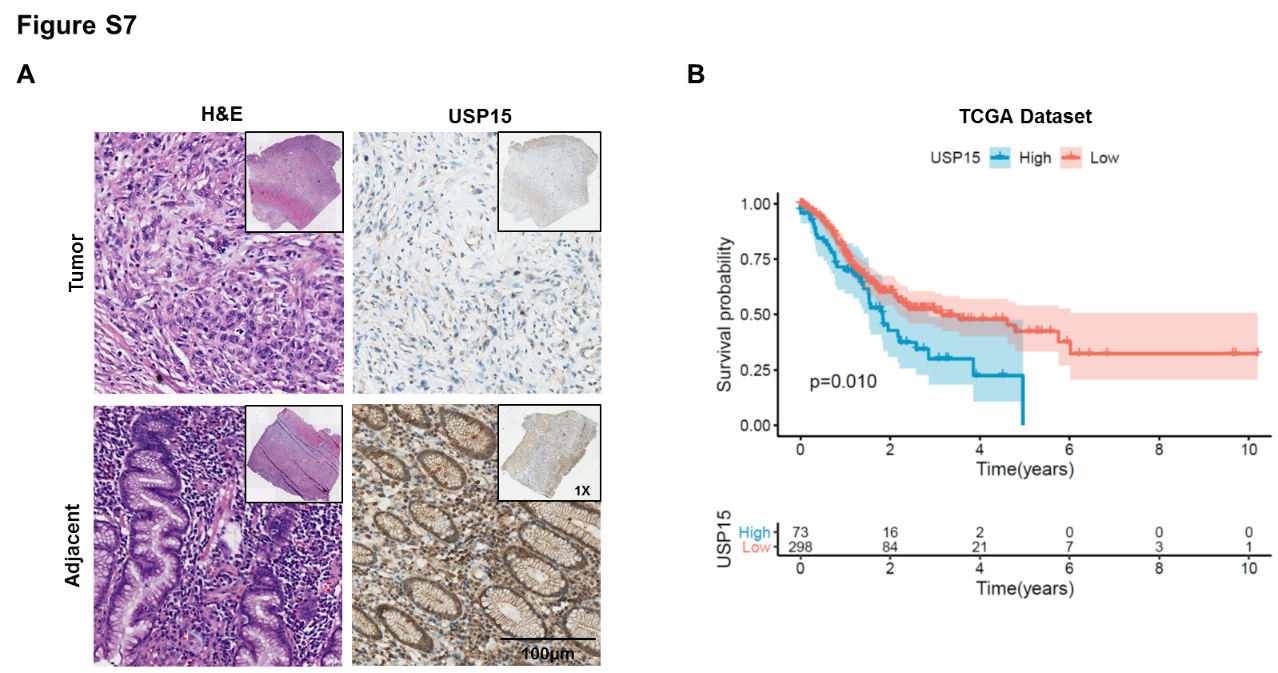


Figure S7. USP15 was downregulated in GC tissues and was associated with poor prognosis of GC patients. (A) H&E staining and IHC staining of USP15 in GC tissues from patients. (B) Kaplan–Meier survival analysis of overall survival obtained from public gene expression datasets.


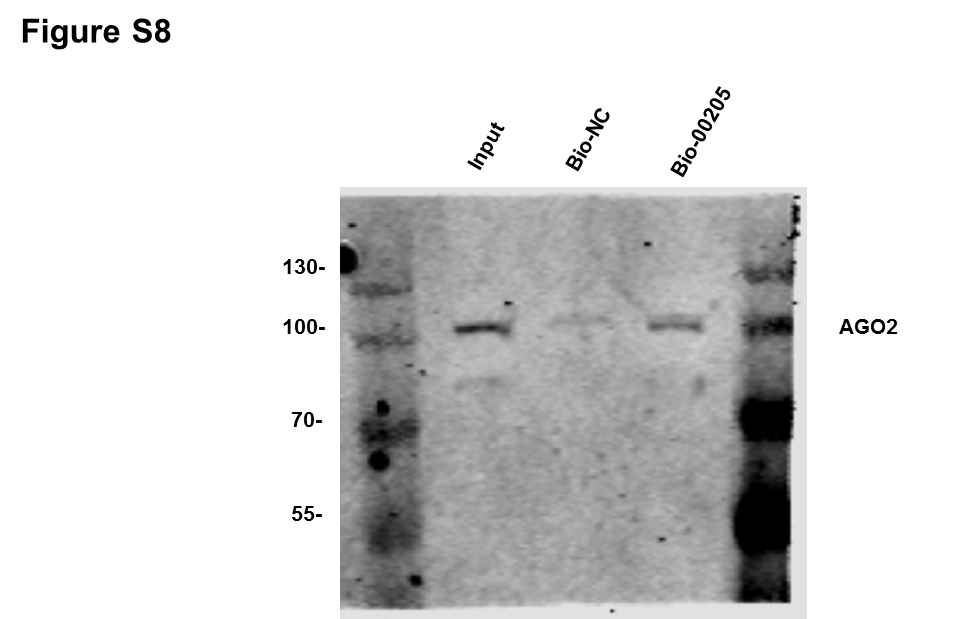


Figure S8. RNA affinity isolation using biotin-labeled LINC00205 probes, and western blot analysis further confirmed the enrichments of AGO2 in the biotin-LINC00205 probe group.

**Supplementary Tables**

Table S1. Pathological information of human gastric tissue samples for lncRNA and mRNA qRT-PCR validation.

| Sample ID | Gender | Age | TNM | Stage | Lauren classification | Histological grade |
| --- | --- | --- | --- | --- | --- | --- |
| 1 | M | 56 | T2N0M0 | IIIB | Intestinal | Moderate |
| 2 | F | 47 | T3N2M0 | IIIB | N/A | Moderate |
| 3 | M | 59 | T3N3M0 | IIIA | Intestinal | Moderate |
| 4 | M | 53 | T2N2M0 | IIIB | Mixed | Poor |
| 5 | M | 55 | T3N3M0 | IIB | Diffuse | Well |
| 6 | M | 56 | T3N2M0 | IIIB | Intestinal | Moderate |
| 7 | F | 57 | T3N0M0 | IIIA | Intestinal | Moderate |
| 8 | F | 74 | T3N0M0 | IIA | Diffuse | Poor |
| 9 | M | 58 | T3N0M0 | IIA | Intestinal | Moderate |
| 10 | M | 55 | T4N1M0 | III | Diffuse | Poor |
| 11 | M | 58 | T3N2M0 | IIIA | Intestinal | Moderate |
| 12 | M | 78 | T2N1M0 | IIA | Mixed | Moderate |
| 13 | F | 50 | T2N3M0 | IIIA | Intestinal | Moderate |
| 14 | F | 32 | T3N3M0 | IIB | Diffuse | Poor |
| 15 | M | 68 | T3N3M0 | IIIB | Mixed | Moderate |
| 16 | M | 41 | T3N2M0 | IIIB | Diffuse | Poor |
| 17 | M | 69 | T3N3M0 | IIB | Diffuse | Poor |
| 18 | F | 60 | T3N2M1 | IIIB | Intestinal | Poor |
| 19 | F | 41 | T3N2M0 | IV | Intestinal | Moderate |
| 20 | F | 56 | T1N0M0 | IIB | Mixed | Poor |
| 21 | M | 75 | T3N1M0 | IIB | Mixed | Moderate |
| 22 | M | 75 | T3N0M0 | III | Intestinal | Moderate |
| 23 | M | 45 | T3N0M0 | IIB | Diffuse | Poor |
| 24 | M | 55 | T1N0M0 | IIIA | Intestinal | Moderate |
| 25 | M | 44 | T3N1M0 | IIIB | Intestinal | Moderate |
| 26 | M | 60 | T3N1M0 | IIB | Intestinal | Moderate |
| 27 | F | 53 | T2N0M0 | IIA | Mixed | Moderate |
| 28 | F | 57 | T3N0M0 | IIIB | Diffuse | Poor |
| 29 | M | 38 | T3N3M0 | IIB | Diffuse | Moderate |
| 30 | F | 56 | T3N0M0 | IIA | Diffuse | Poor |
| 31 | M | 62 | T2N0M0 | IIIB | Intestinal | Moderate |
| 32 | M | 69 | T2N3M0 | IIA | Intestinal | Moderate |
| 33 | F | 48 | T2N0M0 | IIIB | Mixed | Moderate |
| 34 | F | 70 | T2N1M0 | IA | Mixed | Poor |
| 35 | M | 80 | T3N2M0 | IIB | Diffuse | Poor |
| 36 | M | 65 | T3N1M0 | IIB | Mixed | Moderate |
| 37 | M | 61 | T3N0M0 | IIIB | Intestinal | N/A |
| 38 | M | 59 | T3N3M0 | IB | Diffuse | Poor |
| 39 | F | 53 | T3N3M0 | IIA | Mixed | Poor |
| 40 | F | 74 | T3N0M0 | IIIA | Intestinal | Poor |
| 41 | M | 61 | T2N2M0 | IIIB | Intestinal | Poor |
| 42 | M | 72 | T3N1M0 | IIA | Intestinal | Moderate |
| 43 | M | 49 | T3N1M0 | IB | Intestinal | Moderate |
| 44 | F | 64 | T4N3M0 | IB | Diffuse | Poor |
| 45 | F | 58 | T1N0M0 | IIA | Diffuse | Poor |
| 46 | M | 71 | T2N1M0 | IIA | Intestinal | Poor |
| 47 | M | 70 | T2N1M0 | IIIA | Intestinal | N/A |
| 48 | M | 63 | T1N0M0 | IIB | Mixed | Poor |
| 49 | M | 67 | T4N2M0 | IIA | Intestinal | Poor |
| 50 | M | 54 | T4N0M0 | IIIB | N/A | Poor |
| 51 | M | 66 | T4N3M0 | IIIB | N/A | Poor |
| 52 | M | 56 | T3N0M0 | IIA | Mixed | Moderate |
| 53 | F | 55 | T3N0M0 | IIB | Diffuse | Poor |
| 54 | M | 45 | T1N0M0 | IIB | Diffuse | Poor |
| 55 | M | 52 | T3N3M0 | IIB | Intestinal | Moderate |
| 56 | M | 36 | T3N0M0 | III | Diffuse | Poor |
| 57 | M | 60 | T4N2M0 | IA | Diffuse | Poor |
| 58 | M | 51 | T3N3M0 | IIA | Intestinal | Poor |
| 59 | F | 69 | T3N1M0 | IIA | Mixed | Moderate |
| 60 | M | 59 | T3N3M0 | IIA | Intestinal | Well |
| 61 | M | 48 | T3N3M0 | IA | Diffuse | Poor |
| 62 | M | 46 | T4N1M0 | III | Mixed | Poor |
| 63 | F | 41 | T3N2M0 | IIB | Mixed | Moderate |
| 64 | M | 54 | T3N1M0 | IIIB | Intestinal | Moderate |
| 65 | M | 58 | T2N3M0 | III | Diffuse | Poor |
| 66 | M | 55 | T3N2M0 | IIA | Diffuse | Poor |
| 67 | M | 62 | T3N2M0 | IIA | Intestinal | Moderate |
| 68 | M | 51 | T2N0M0 | IIIB | Intestinal | Moderate |
| 69 | F | 68 | T4N2M0 | IIIB | Intestinal | Moderate |
| 70 | M | 42 | T3N2M0 | IIA | Intestinal | Moderate |
| 71 | F | 53 | T3N2M0 | IIA | Diffuse | Poor |
| 72 | M | 52 | T3N1M0 | III | Intestinal | Moderate |
| 73 | F | 34 | T2N0M0 | IIIB | N/A | Moderate |
| 74 | M | 49 | T3N3M0 | IIB | N/A | Moderate |
| 75 | M | 71 | T3N0M0 | IIIB | N/A | Moderate |
| 76 | M | 61 | T2N2M0 | IIIB | N/A | Moderate |
| 77 | F | 58 | T3N2M0 | III | N/A | Moderate |
| 78 | F | 67 | T2N2M0 | IIIA | Intestinal | Moderate |
| 79 | M | 59 | T3N0M0 | IIIB | Intestinal | Moderate |
| 80 | F | 52 | T3N1M0 | IIB | Intestinal | Moderate |
| 81 | M | 82 | T2N3M0 | IIB | Diffuse | Poor |
| 82 | F | 52 | T4N1M0 | III | Intestinal | Moderate |
| 83 | F | 52 | T3N2M0 | IIIB | Intestinal | Moderate |
| 84 | M | 69 | T3N0M0 | IB | Intestinal | Moderate |
| 85 | F | 79 | T2N0M0 | IIB | Intestinal | Poor |
| 86 | M | 58 | T3N3M0 | IIB | Mixed | Poor |
| 87 | F | 64 | T3N3M0 | IB | Mixed | Moderate |
| 88 | M | 52 | T3N0M0 | IIA | Intestinal | Moderate |
| 89 | M | 60 | T4N2M0 | IIIA | N/A | Moderate |
| 90 | F | 71 | T3N2M0 | IA | Mixed | Moderate |
| 91 | F | 81 | T4N3M1 | IIIB | Mixed | Moderate |
| 92 | M | 74 | T2N1M0 | IIIA | Intestinal | Moderate |
| 93 | F | 62 | T2N3M0 | IIA | Diffuse | Poor |
| 94 | M | 47 | T2N0M0 | IIIB | Diffuse | Poor |
| 95 | F | 71 | T2N0M0 | IA | Mixed | Poor |
| 96 | M | 69 | T3N0M0 | IIIA | Diffuse | Poor |
| 97 | M | 63 | T3N3M0 | IIIA | Intestinal | Moderate |
| 98 | M | 57 | T3N3M0 | IIIA | Mixed | Moderate |
| 99 | M | 50 | T3N1M0 | IB | Mixed | Moderate |
| 100 | M | 57 | T3N2M0 | III | Mixed | Poor |
| 101 | M | 64 | T4N2M0 | IIIA | Intestinal | Moderate |
| 102 | M | 71 | T2N0M0 | IIIA | Mixed | Poor |
| 103 | M | 33 | T3N2M0 | IB | Mixed | Poor |
| 104 | M | 69 | T3N0M0 | IIIB | Intestinal | Moderate |
| 105 | M | 56 | T3N3M0 | IIA | Intestinal | Moderate |
| 106 | M | 42 | T3N0M0 | IIIA | Mixed | Moderate |
| 107 | F | 64 | T3N3M0 | IIB | Intestinal | Moderate |

Table S2. Pathological information of blood samples for miRNA qRT-PCR validation.

| Sample ID | Gender | Age | Pathology/diagnosis |
| --- | --- | --- | --- |
| 1 | M | 68 | Adenocarcinoma |
| 2 | M | 52 | Adenocarcinoma |
| 3 | F | 33 | Adenocarcinoma |
| 4 | M | 71 | Adenocarcinoma |
| 5 | M | 54 | Adenocarcinoma |
| 6 | M | 68 | Adenocarcinoma |
| 7 | M | 57 | Adenocarcinoma |
| 8 | M | 64 | Adenocarcinoma |
| 9 | M | 57 | Adenocarcinoma |
| 10 | M | 64 | Adenocarcinoma |
| 11 | F | 57 | Adenocarcinoma |
| 12 | M | 65 | Adenocarcinoma |
| 13 | F | 67 | Adenocarcinoma |
| 14 | M | 53 | Adenocarcinoma |
| 15 | M | 69 | Adenocarcinoma |
| 16 | M | 50 | Hepatic metastases from GC |
| 17 | F | 74 | Adenocarcinoma |
| 18 | M | 71 | Adenocarcinoma |
| 19 | M | 72 | Adenocarcinoma |
| 20 | M | 69 | Suspected hepatoid adenocarcinoma |
| 21 | M | 58 | Adenocarcinoma |
| 22 | M | 56 | Normal |
| 23 | M | 61 | Normal |
| 24 | F | 65 | Normal |
| 25 | F | 60 | Normal |
| 26 | F | 55 | Normal |
| 27 | M | 48 | Normal |
| 28 | M | 67 | Normal |
| 29 | M | 68 | Normal |
| 30 | F | 70 | Normal |
| 31 | M | 66 | Normal |
| 32 | M | 54 | Normal |
| 33 | M | 55 | Normal |
| 34 | M | 49 | Normal |
| 35 | M | 60 | Normal |
| 36 | F | 51 | Normal |
| 37 | F | 47 | Normal |
| 38 | M | 62 | Normal |
| 39 | F | 54 | Normal |
| 40 | M | 53 | Normal |
| 41 | F | 49 | Normal |
| 42 | M | 53 | Normal |
| 43 | M | 51 | Normal |

Table S3. LncRNAs affinity-isolation probe sequences.

| Probe | Sequence (5’--------------3’) |
| --- | --- |
| LINC00205 | TTCTAGGAGACGAGAGAGCGAACGCCAGGGACCCTGTGCAGGCCTGCTCCTCCGTTTGCAAGGTGAGTTACCAGGTTCACGTGTTTGGAGTTTCTGGACTCATTGCGGAGTTCCACCCCTGCACGTTGCGGTTCCCCAGTAATCAAATCCTGGCTTTTGTGCCTGGAAGTGCACAGGGAGGGGACAACTTTGTGAGTCAGTGGCAGGGCAGGGAGTTCTGGTTCTCCAGAGCCAGAGGCCGTGCTCAGAAGGATTTCTTAGCAGGAGCCTTGGGGCCCCCAGTCAACACTTCCCTACGGACAGCCTGGCCAGCTCTGTGCACGGAGCAGGCGCCCGAGGGTCCCAGGTCACCAAGTGACCAAGTCGTGAAGGCGCCCAGGGTTTCCTGGGGGTGCGCTGATCCCAAGGAAGCCACGTGTGGTCAGCATGGGGGAGGGGACCAGCGCCCCGGGGGGCCTGCAGCACAGCAGGGCCTGCCCTCCTGGGTGAGACTGGCCGGTGCCTGTGGGGATCTGGGGGGCTACAGTCAGGGCTCTGTGCTCCCGAGGGCCACGCCAGCCCACCTGCCCTGGAACAGAACCCGAGGCTTCTGCCTAGGGGAGTACCTGGGCACCTGCCTCCTGTG |
| Negative  Control | CTTGGTACCGAGCTCGGATCCACTAGTCCAGTGTGGTGGAATTCTGCAGATATCCAGCACAGTGGCGGCCG |

Table S4. Primers list for miRNA and mRNA qRT-PCR validation.

| Primer |  | Sequence (5’--------------3’) |
| --- | --- | --- |
| LINC00205 | Forward | TTGAGACGGGAGTGTTCAGC |
|  | Reverse | TCACTGGAGAGGGAGACGAG |
| hsa-miR-26a | Forward | ACACTCCAGCTGGGTTCAAGTAATCCAGGA |
|  | Reverse | TGGTGTCGTGGAGTCG |
| USP15 | Forward | CGACGCTGCTCAAAACCTC |
|  | Reverse | TCCCATCTGGTATTTGTCCCAA |
| HMGA2 | Forward | ACCCAGGGGAAGACCCAAA |
|  | Reverse | CCTCTTGGCCGTTTTTCTCCA |
| EZH2 | Forward | AATCAGAGTACATGCGACTGAGA |
|  | Reverse | GCTGTATCCTTCGCTGTTTCC |
| U6 | Forward | CTCGCTTCGGCAGCACATATACT |
|  | Reverse | ACGCTTCACGAATTTGCGTGTC |
| GAPDH | Forward | GACTCATGACCACAGTCCATGC |
|  | Reverse | AGAGGCAGGGATGAT GTTCTG |
