# Supplementary material for: Novel prognostic marker LINC00205 promotes tumorigenesis and metastasis by competitively suppressing miRNA-26a in gastric cancer
Source: Cell Death Discov. 2022 Jan 10;8:5. doi: 10.1038/s41420-021-00802-8 (PMC8748761; doi:10.1038/s41420-021-00802-8)
Supplement: Supplementary file 2 — all of the co-authors’ consent letters [file 41420_2021_802_MOESM2_ESM.pdf]

Fw: Re: Author confirmation request

ydlongtao@bjmu.edu.cn

芳

邢晓芳 2021-12-11 20:31

发至 我

详情

-----原始邮件-----

发件人:huangfu <ydlongtao@bjmu.edu.cn>

发送时间:2021-12-10 10:50:39 (星期五)

收件人: "xingxiaofang@bjmu.edu.cn" <xingxiaofang@bjmu.edu.cn>

抄送:

主题: Re: Author confirmation request

I confirmed

ydlongtao

ydlongtao@bjmu.edu.cn

签名由 网易邮箱大师 定制

On 12/10/2021 10:22，邢晓芳<xingxiaofang@bjmu.edu.cn> wrote：

You are receiving this email because you have been listed as an author on a manuscript recently provisionally accepted for publication in Cell Death Discovery. The manuscript details are below.

Title: Novel prognostic marker LINC00205 promotes tumorigenesis and metastasis by competitively suppressing miRNA-26a in gastric cancer

Authors: Longtao Huangfu, Biao Fan, Gangjian Wang, Xuejun Gan, Shanshan Tian, Qifei He, Qian Yao, Jin yao Shi, Xiaomei Li, Hong Du, Xiangyu Gao, Xiaofang Xing, Jiafu Ji

Corresponding author: Jiafu Ji, Xiaofang Xing

Please reply to my email confirming that you agree to the author contributions and the final author list in this manuscript .

Fw: Re:Author confirmation request

ydlongtao@bjmu.edu.cn

芳

邢晓芳 2021-12-11 20:31
发至 我

详情

-----原始邮件-----
发件人:"Biao Fan" <fanbiao1986@163.com>
发送时间:2021-12-10 22:51:21 (星期五)
收件人: "邢晓芳" <xingxiaofang@bjmu.edu.cn>
抄送:
主题: Re:Author confirmation request

Dear Prof. Xing,
Yes, I agree to the author contributions and the final author list in this manuscript.
Best wishes,
Biao Fan

At 2021-12-10 10:22:14, "邢晓芳" <xingxiaofang@bjmu.edu.cn> wrote:

> You are receiving this email because you have been listed as an author on a manuscript recently provisionally accepted for publication in Cell Death Discovery. The manuscript details are below.
>
> Title: Novel prognostic marker LINC00205 promotes tumorigenesis and metastasis by competitively suppressing miRNA-26a in gastric cancer
>
> Authors: Longtao Huangfu, Biao Fan, Gangjian Wang, Xuejun Gan, Shanshan Tian, Qifei He, Qian Yao, Jin yao Shi, Xiaomei Li, Hong Du, Xiangyu Gao, Xiaofang Xing, Jiafu Ji
>
> Corresponding author: Jiafu Ji, Xiaofang Xing
>
> Please reply to my email confirming that you agree to the author contributions and the final author list in this manuscript .

-----原始邮件-----
发件人:"Gangjian Wang" <gangjian_wang1@163.com>
发送时间:2021-12-10 11:01:13 (星期五)
收件人: "xingxiaofang@bjmu.edu.cn" <xingxiaofang@bjmu.edu.cn>
抄送:
主题: Re: Author confirmation request

confirmed!

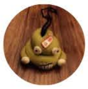

Gangjian Wang

gangjian_wang1@163.com

签名由 网易邮箱大师 定制

On 12/10/2021 10:29，Gangjian Wang<gangjian_wang1@163.com> wrote：

收到

---- 回复的原邮件 ----

At 2021-12-10 10:22:14, "邢晓芳" <xingxiaofang@bjmu.edu.cn> wrote:

> You are receiving this email because you have been listed as an author on a manuscript recently provisionally accepted for publication in Cell Death Discovery. The manuscript details are below.
>
> Title: Novel prognostic marker LINC00205 promotes tumorigenesis and metastasis by competitively suppressing miRNA-26a in gastric cancer
>
> Authors: Longtao Huangfu, Biao Fan, Gangjian Wang, Xuejun Gan, Shanshan Tian, Qifei He, Qian Yao, Jin yao Shi, Xiaomei Li, Hong Du, Xiangyu Gao, Xiaofang Xing, Jiafu Ji
>
> Corresponding author: Jiafu Ji, Xiaofang Xing
>
> Please reply to my email confirming that you agree to the author contributions and the final author list in this manuscript .

Fw: Re:Author confirmation request

ydlongtao@bjmu.edu.cn

芳

邢晓芳 2021-12-10 16:59

发至 我

详情

-----原始邮件-----

发件人:"甘雪君" <ggxjiujiu@163.com>

发送时间:2021-12-10 11:02:08 (星期五)

收件人: "邢晓芳" <xingxiaofang@bjmu.edu.cn>

抄送:

主题: Re:Author confirmation request

confirmed

At 2021-12-10 10:22:14, "邢晓芳" <xingxiaofang@bjmu.edu.cn> wrote:

> You are receiving this email because you have been listed as an author on a manuscript recently provisionally accepted for publication in Cell Death Discovery. The manuscript details are below.
>
> Title: Novel prognostic marker LINC00205 promotes tumorigenesis and metastasis by competitively suppressing miRNA-26a in gastric cancer
>
> Authors: Longtao Huangfu, Biao Fan, Gangjian Wang, Xuejun Gan, Shanshan Tian, Qifei He, Qian Yao, Jin yao Shi, Xiaomei Li, Hong Du, Xiangyu Gao, Xiaofang Xing, Jiafu Ji
>
> Corresponding author: Jiafu Ji, Xiaofang Xing
>
> Please reply to my email confirming that you agree to the author contributions and the final author list in this manuscript .

-----原始邮件-----
发件人:"田姗姗" <tianshanshan@bjmu.edu.cn>
发送时间:2021-12-10 15:37:12 (星期五)
收件人: "邢晓芳" <xingxiaofang@bjmu.edu.cn>
抄送:
主题: Re: Author confirmation request

I confirmed

> -----原始邮件-----
> **发件人:**"邢晓芳" <xingxiaofang@bjmu.edu.cn>
> **发送时间:**2021-12-10 10:22:14 (星期五)
> **收件人:** ydlongtao@bjmu.edu.cn, fanbiao1986@163.com, gangjian_wang1@163.com, ggxjiujiu@163.com, tianshanshan@bjmu.edu.cn, dr_chiephy@hsc.pku.edu.cn, yaoqian@bjmu.edu.cn, shijinyao@bjmu.edu.cn, lixiaomei1984@sina.cn, dhdh-69@163.com, xiangyugao@hsc.pku.edu.cn
> **抄送:**
> **主题:** Author confirmation request
>
> You are receiving this email because you have been listed as an author on a manuscript recently provisionally accepted for publication in Cell Death Discovery. The manuscript details are below.
>
> Title: Novel prognostic marker LINC00205 promotes tumorigenesis and metastasis by competitively suppressing miRNA-26a in gastric cancer
>
> Authors: Longtao Huangfu, Biao Fan, Gangjian Wang, Xuejun Gan, Shanshan Tian, Qifei He, Qian Yao, Jin yao Shi, Xiaomei Li, Hong Du, Xiangyu Gao, Xiaofang Xing, Jiafu Ji
>
> Corresponding author: Jiafu Ji, Xiaofang Xing
>
> Please reply to my email confirming that you agree to the author contributions and the final author list in this manuscript .

-----原始邮件-----
发件人:"Qifei He" <dr_chiephy@bjmu.edu.cn>
发送时间:2021-12-10 11:00:27 (星期五)
收件人: "邢晓芳" <xingxiaofang@bjmu.edu.cn>
抄送:
主题: Re: Author confirmation request

Thanks for your remind. I agree to the author contributions and the final author list in this manuscript.

-----原始邮件-----
发件人:"邢晓芳" <xingxiaofang@bjmu.edu.cn>
发送时间:2021-12-10 10:22:14 (星期五)
收件人: ydlongtao@bjmu.edu.cn, fanbiao1986@163.com, gangjian_wang1@163.com, ggxjiujiu@163.com, tianshanshan@bjmu.edu.cn, dr_chiephy@hsc.pku.edu.cn, yaoqian@bjmu.edu.cn, shijinyao@bjmu.edu.cn, lixiaomei1984@sina.cn, dhdh-69@163.com, xiangyugao@hsc.pku.edu.cn
抄送:
主题: Author confirmation request

You are receiving this email because you have been listed as an author on a manuscript recently provisionally accepted for publication in Cell Death Discovery. The manuscript details are below.

Title: Novel prognostic marker LINC00205 promotes tumorigenesis and metastasis by competitively suppressing miRNA-26a in gastric cancer

Authors: Longtao Huangfu, Biao Fan, Gangjian Wang, Xuejun Gan, Shanshan Tian, Qifei He, Qian Yao, Jin yao Shi, Xiaomei Li, Hong Du, Xiangyu Gao, Xiaofang Xing, Jiafu Ji

Corresponding author: Jiafu Ji, Xiaofang Xing

Please reply to my email confirming that you agree to the author contributions and the final author list in this manuscript .

-----原始邮件-----
发件人:"姚倩" <yaoqian@bjmu.edu.cn>
发送时间:2021-12-10 10:56:26 (星期五)
收件人: "邢晓芳" <xingxiaofang@bjmu.edu.cn>
抄送:
主题: Re: Author confirmation request

Confirmed

> -----原始邮件-----
> **发件人:**"邢晓芳" <xingxiaofang@bjmu.edu.cn>
> **发送时间:**2021-12-10 10:22:14 (星期五)
> **收件人:** ydlongtao@bjmu.edu.cn, fanbiao1986@163.com, gangjian_wang1@163.com, ggxjiujiu@163.com, tianshanshan@bjmu.edu.cn, dr_chiephy@hsc.pku.edu.cn, yaoqian@bjmu.edu.cn, shijinyao@bjmu.edu.cn, lixiaomei1984@sina.cn, dhdh-69@163.com, xiangyugao@hsc.pku.edu.cn
> **抄送:**
> **主题:** Author confirmation request
>
> You are receiving this email because you have been listed as an author on a manuscript recently provisionally accepted for publication in Cell Death Discovery. The manuscript details are below.
>
> Title: Novel prognostic marker LINC00205 promotes tumorigenesis and metastasis by competitively suppressing miRNA-26a in gastric cancer
>
> Authors: Longtao Huangfu, Biao Fan, Gangjian Wang, Xuejun Gan, Shanshan Tian, Qifei He, Qian Yao, Jin yao Shi, Xiaomei Li, Hong Du, Xiangyu Gao, Xiaofang Xing, Jiafu Ji
>
> Corresponding author: Jiafu Ji, Xiaofang Xing
>
> Please reply to my email confirming that you agree to the author contributions and the final author list in this manuscript .

-----原始邮件-----

发件人:"石晋瑶" <shijinyao@bjmu.edu.cn>

发送时间:2021-12-10 11:05:51 (星期五)

收件人: "邢晓芳" <xingxiaofang@bjmu.edu.cn>

抄送:

主题: Re: Author confirmation request

Jinyao Shi confirmed

-----原始邮件-----

发件人:"邢晓芳" <xingxiaofang@bjmu.edu.cn>

发送时间:2021-12-10 10:22:14 (星期五)

收件人: ydlongtao@bjmu.edu.cn, fanbiao1986@163.com, gangjian_wang1@163.com, ggxjiujiu@163.com, tianshanshan@bjmu.edu.cn, dr_chiephy@hsc.pku.edu.cn, yaoqian@bjmu.edu.cn, shijinyao@bjmu.edu.cn, lixiaomei1984@sina.cn, dhdh-69@163.com, xiangyugao@hsc.pku.edu.cn

抄送:

主题: Author confirmation request

You are receiving this email because you have been listed as an author on a manuscript recently provisionally accepted for publication in Cell Death Discovery. The manuscript details are below.

Title: Novel prognostic marker LINC00205 promotes tumorigenesis and metastasis by competitively suppressing miRNA-26a in gastric cancer

Authors: Longtao Huangfu, Biao Fan, Gangjian Wang, Xuejun Gan, Shanshan Tian, Qifei He, Qian Yao, Jin yao Shi, Xiaomei Li, Hong Du, Xiangyu Gao, Xiaofang Xing, Jiafu Ji

Corresponding author: Jiafu Ji, Xiaofang Xing

Please reply to my email confirming that you agree to the author contributions and the final author list in this manuscript .

Fw: 回复：Author confirmation request

ydlongtao@bjmu.edu.cn

芳

邢晓芳 2021-12-10 17:54
发至 我

详情

-----原始邮件-----
发件人:lixiaomei1984@sina.cn
发送时间:2021-12-10 17:06:32 (星期五)
收件人: Xingxiaofang <xingxiaofang@bjmu.edu.cn>
抄送:
主题: 回复：Author confirmation request

I confirm.

祝您工作顺利，心情愉快！
李小梅（15210085227）
2021.12.10

----- 原始邮件 -----
发件人：邢晓芳 <xingxiaofang@bjmu.edu.cn>
收件人：ydlongtao@bjmu.edu.cn, fanbiao1986@163.com, gangjian_wang1@163.com, ggxjiujiu@163.com, tianshanshan@bjmu.edu.cn, dr_chiephy@hsc.pku.edu.cn, yaoqian@bjmu.edu.cn, shijinyao@bjmu.edu.cn, lixiaomei1984@sina.cn, dhdh-69@163.com, xiangyugao@hsc.pku.edu.cn
主题：Author confirmation request
日期：2021年12月10日 10点22分

You are receiving this email because you have been listed as an author on a manuscript recently provisionally accepted for publication in Cell Death Discovery. The manuscript details are below.

Title: Novel prognostic marker LINC00205 promotes tumorigenesis and metastasis by competitively suppressing miRNA-26a in gastric cancer

Authors: Longtao Huangfu, Biao Fan, Gangjian Wang, Xuejun Gan, Shanshan Tian, Qifei He, Qian Yao, Jin yao Shi, Xiaomei Li, Hong Du, Xiangyu Gao, Xiaofang Xing, Jiafu Ji

Fw: Re:Author confirmation request

ydlongtao@bjmu.edu.cn

芳

邢晓芳 2021-12-10 16:59

发至 我

详情

-----原始邮件-----

发件人:"杜红" <dhdh-69@163.com>

发送时间:2021-12-10 12:32:42 (星期五)

收件人: "邢晓芳" <xingxiaofang@bjmu.edu.cn>

抄送:

主题: Re:Author confirmation request

I confirmed

At 2021-12-10 10:22:14, "邢晓芳" <xingxiaofang@bjmu.edu.cn> wrote:

> You are receiving this email because you have been listed as an author on a manuscript recently provisionally accepted for publication in Cell Death Discovery. The manuscript details are below.
>
> Title: Novel prognostic marker LINC00205 promotes tumorigenesis and metastasis by competitively suppressing miRNA-26a in gastric cancer
>
> Authors: Longtao Huangfu, Biao Fan, Gangjian Wang, Xuejun Gan, Shanshan Tian, Qifei He, Qian Yao, Jin yao Shi, Xiaomei Li, Hong Du, Xiangyu Gao, Xiaofang Xing, Jiafu Ji
>
> Corresponding author: Jiafu Ji, Xiaofang Xing
>
> Please reply to my email confirming that you agree to the author contributions and the final author list in this manuscript .

Fw: Re: Author confirmation request

ydlongtao@bjmu.edu.cn

芳

邢晓芳 2021-12-10 16:59

发至 我

详情

-----原始邮件-----

发件人:"Xiangyu Gao" <xiangyugao@hsc.pku.edu.cn>

发送时间:2021-12-10 11:09:25 (星期五)

收件人: "邢晓芳" <xingxiaofang@bjmu.edu.cn>

抄送:

主题: Re: Author confirmation request

Confirmed.

发自我的iPhone

> 在 2021年12月10日，10:22，邢晓芳 <xingxiaofang@bjmu.edu.cn> 写道:
>
> You are receiving this email because you have been listed as an author on a manuscript recently provisionally accepted for publication in Cell Death Discovery. The manuscript details are below.
>
> Title: Novel prognostic marker LINC00205 promotes tumorigenesis and metastasis by competitively suppressing miRNA-26a in gastric cancer
>
> Authors: Longtao Huangfu, Biao Fan, Gangjian Wang, Xuejun Gan, Shanshan Tian, Qifei He, Qian Yao, Jin yao Shi, Xiaomei Li, Hong Du, Xiangyu Gao, Xiaofang Xing, Jiafu Ji
>
> Corresponding author: Jiafu Ji, Xiaofang Xing
>
> Please reply to my email confirming that you agree to the author contributions and the final author list in this manuscript .

-----原始邮件-----
发件人:"季加孚" <jijiafu@hsc.pku.edu.cn>
发送时间:2021-12-10 16:51:59 (星期五)
收件人: "邢晓芳" <xingxiaofang@bjmu.edu.cn>
抄送:
主题: Re: Author confirmation request

confirmed

> -----原始邮件-----
> 发件人:"邢晓芳" <xingxiaofang@bjmu.edu.cn>
> 发送时间:2021-12-10 16:50:31 (星期五)
> 收件人: jijiafu@hsc.pku.edu.cn
> 抄送:
> 主题: Author confirmation request
>
> You are receiving this email because you have been listed as an author on a manuscript recently provisionally accepted for publication in Cell Death Discovery. The manuscript details are below.
>
> Title: Novel prognostic marker LINC00205 promotes tumorigenesis and metastasis by competitively suppressing miRNA-26a in gastric cancer
>
> Authors: Longtao Huangfu, Biao Fan, Gangjian Wang, Xuejun Gan, Shanshan Tian, Qifei He, Qian Yao, Jin yao Shi, Xiaomei Li, Hong Du, Xiangyu Gao, Xiaofang Xing, Jiafu Ji
>
> Corresponding author: Jiafu Ji, Xiaofang Xing
>
> Please reply to my email confirming that you agree to the author contributions and the final author list in this manuscript .

Jiafu Ji, MD, PhD, FACS, FRCS
President of Peking University Cancer Hospital

TEL +86-10-88196048(W)
E-mail: jiafuji@outlook.com; jijiafu@hsc.pku.edu.cn
